# Bacteria primed by antimicrobial peptides develop tolerance and persist

**DOI:** 10.1371/journal.ppat.1009443

**Published:** 2021-03-31

**Authors:** Alexandro Rodríguez-Rojas, Desiree Y. Baeder, Paul Johnston, Roland R. Regoes, Jens Rolff

**Affiliations:** 1 Freie Universität Berlin, Institut für Biologie, Evolutionary Biology, Berlin, Germany; 2 Institute of Integrative Biology, Zürich, Switzerland; 3 Berlin Center for Genomics in Biodiversity Research, Berlin, Germany; 4 Leibniz-Institute of Freshwater Ecology and Inland Fisheries (IGB), Berlin, Germany; 5 Berlin-Brandenburg Institute of Advanced Biodiversity Research (BBIB), Berlin, Germany; University of Toronto, CANADA

## Abstract

Antimicrobial peptides (AMPs) are key components of innate immune defenses. Because of the antibiotic crisis, AMPs have also come into focus as new drugs. Here, we explore whether prior exposure to sub-lethal doses of AMPs increases bacterial survival and abets the evolution of resistance. We show that *Escherichia coli* primed by sub-lethal doses of AMPs develop tolerance and increase persistence by producing curli or colanic acid, responses linked to biofilm formation. We develop a population dynamic model that predicts that priming delays the clearance of infections and fuels the evolution of resistance. The effects we describe should apply to many AMPs and other drugs that target the cell surface. The optimal strategy to tackle tolerant or persistent cells requires high concentrations of AMPs and fast and long-lasting expression. Our findings also offer a new understanding of non-inherited drug resistance as an adaptive response and could lead to measures that slow the evolution of resistance.

## Author summary

Animals and plants defend themselves with ancient molecules called antimicrobial peptides (AMPs) against pathogens. As more and more bacterial diseases have become drug resistant, these AMPs are considered as promising alternatives. In natural situation such as on the skin, bacteria are often exposed to low concentrations of AMPs that do no kill. Here we show that the bacterium *Escherichia coli* when exposed to such low concentrations becomes recalcitrant to killing concentrations of the same AMPs. We report the ways in which the bacteria alter their surface to do so. We then use a mathematical model to show that these effects caused by low concentrations can drive the evolution of resistance. From the perspective of an organism using AMPs in self-defense, the best option is to deploy high concentrations of AMPs for long. Our findings also offer a new understanding of similar drug resistance mechanisms.

## Introduction

Antimicrobial peptides—short, usually cationic molecules—are key effectors of innate immune defences of all multicellular life [1] and are also important players at the host microbiota interface [2,3]. Because of their evolutionary success and diversity, AMPs are considered as new antimicrobial drugs to alleviate the antibiotic resistance crisis [4] with more than two dozen currently under clinical trial [5]. *Bona fide* genetic resistance of bacteria towards AMPs has been studied [6,7], but not to the extent of antibiotic resistance. Resistance against AMPs evolves usually with a low probability and the levels of resistance are not as high as against antibiotics [8,9].

Notably, non-inherited resistance [10], the ability of bacteria to survive lethal concentrations of antimicrobials without a genetically encoded resistance mechanism, has rarely been studied for AMPs. One of the few studies that has addressed non-inherited resistance in a natural host-microbe interaction is the example of the bobtail squid and its symbiont *Vibrio fischeri*. Here, non-inherited resistance is elicited by a low pH and primes *V*. *fischeri* to colonize the light-emitting organ of the squid [11] in the presence of high concentrations of AMPs. This results from the bacteria’s overlapping stress responses to AMP exposition and acidic pH.

Microbes have evolved adaptive physiological alterations to predictable environmental changes [12,13]. This has been studied in the context of available carbon sources during gut passage of *E*. *coli*, where bacteria respond to a drop in oxygen by switching the genes required for the utilization of different carbon sources [12]. A meta-analysis found that priming, the phenotypic response to a low level stressor, provides a fitness benefit for microbes against a variety of stressors including pH, temperature and oxidative stress [14].

Here, we ask, since antimicrobials frequently occur at sub-inhibitory concentrations, whether a previous encounter with a sub-lethal dose of AMPs induces bacterial tolerance or persistence. For example, upon infection, native AMPs are first induced at sub-lethal concentrations and it usually takes a few hours for them to reach high concentrations [15]. Such pharmacokinetic profiles are mirrored in many antimicrobial drug treatments, whereby during medical application of antibiotics, the pharmacokinetics start at zero and the killing concentrations build up over time.

Non-inherited resistance can be induced by sublethal levels of antimicrobial drugs and peptides [10]. Non-inherited resistance describes phenomena where bacteria are phenotypically refractory to killing concentrations of antimicrobials: the two main mechanisms are tolerance and persistence [16]. Tolerance is defined as an extended time to killing, usually measured by the minimum duration to kill 99% of the population (MDK_99_) while persistence relates to a subpopulation of cells with an extended MDK_99,_ in which case the population is heterogeneous [16]. Persister cells are presumed to be dormant cells with low metabolic activity [17]. Hence they are refractory to killing by bactericidal drugs and can recover metabolic activity and resume growth and colonization once that the drug concentration has dropped to sub-lethal concentrations [18]. Neither tolerant nor persistent cells show an increased MIC (minimum inhibitory concentration). Non-inherited resistance resulting in either drug tolerance or persister cell formation (see S1 Fig) [16] has been shown to be of great importance to understand antibiotic resistance and treatment failure [10,16,19,20]. It can also facilitate *bona fide* resistance evolution against antibiotics [20]. We adopt these findings and concepts from antibiotic research to understand resistance against AMPs, an insight that should equally inform our understanding of host-microbe interactions as well as resistance evolution against AMPs as drugs. It is noteworthy that AMPs differ significantly from conventional antibiotics in several aspects including their pharmacodynamics, resulting in narrower mutant selection windows [9]. In addition, the speed at which they kill bacterial cells is often very fast, within minutes [21] rather than within hours, as it is the case for antibiotics [22]. There are some situations where AMPs kill slowly: *Mycobacterium tuberculosis*, a slow growing bacterium, is killed within hours by the human neutrophil defensin HNP-1 [23]. Notably, anti-tuberculosis antibiotic drugs take days to kill [24]. Finally, AMP killing does not elicit stress responses such as those mediated by the Sigma S(RpoS) and the SOS response, the latter being an important elicitor of persister formation under antibiotic stress [17].

In this study, we specifically investigate whether prior exposure to sub-lethal doses of AMPs increases bacterial survival via either tolerance or persistence and alters the risk of resistance evolution. We use two antimicrobial peptides that are well characterised as a case study. Melittin is a 26 amino acid residue linear peptide from the honeybee, which kills bacterial cells by permeabilisation of the membrane [25]. Melittin is also active against eukaryotic parasites such as *Leishmania* and cancer cells [26]. The second AMP, pexiganan, also a linear peptide, is the first eukaryotic AMP developed as a drug mainly to treat foot ulcers. It is a synthetic 22 amino acid residue peptide closely related to magainin II from the African clawed frog [27]. It shows a broad activity against both, Gram^+^ and Gram^-^ bacteria and kills them by forming toroidal pores [27]. We combine *in vitro* experiments with a modelling approach to study priming and resistance emergence. We find that a sub-lethal dose of the AMPs melittin and pexiganan can induce increased tolerance and/or persistence in bacteria and hence prime [28] them for exposure to a subsequent lethal dose. We also identify candidate underlying molecular mechanisms and capture the population dynamics by adapting a classic mathematical model of persistence [29]. With computer simulations, we then predict that increasing tolerance and persistence will have a positive effect on bacterial survival and the emergence of resistance.

## Results

### Primed cells are less susceptible to killing by AMPs

**Fig 1:**
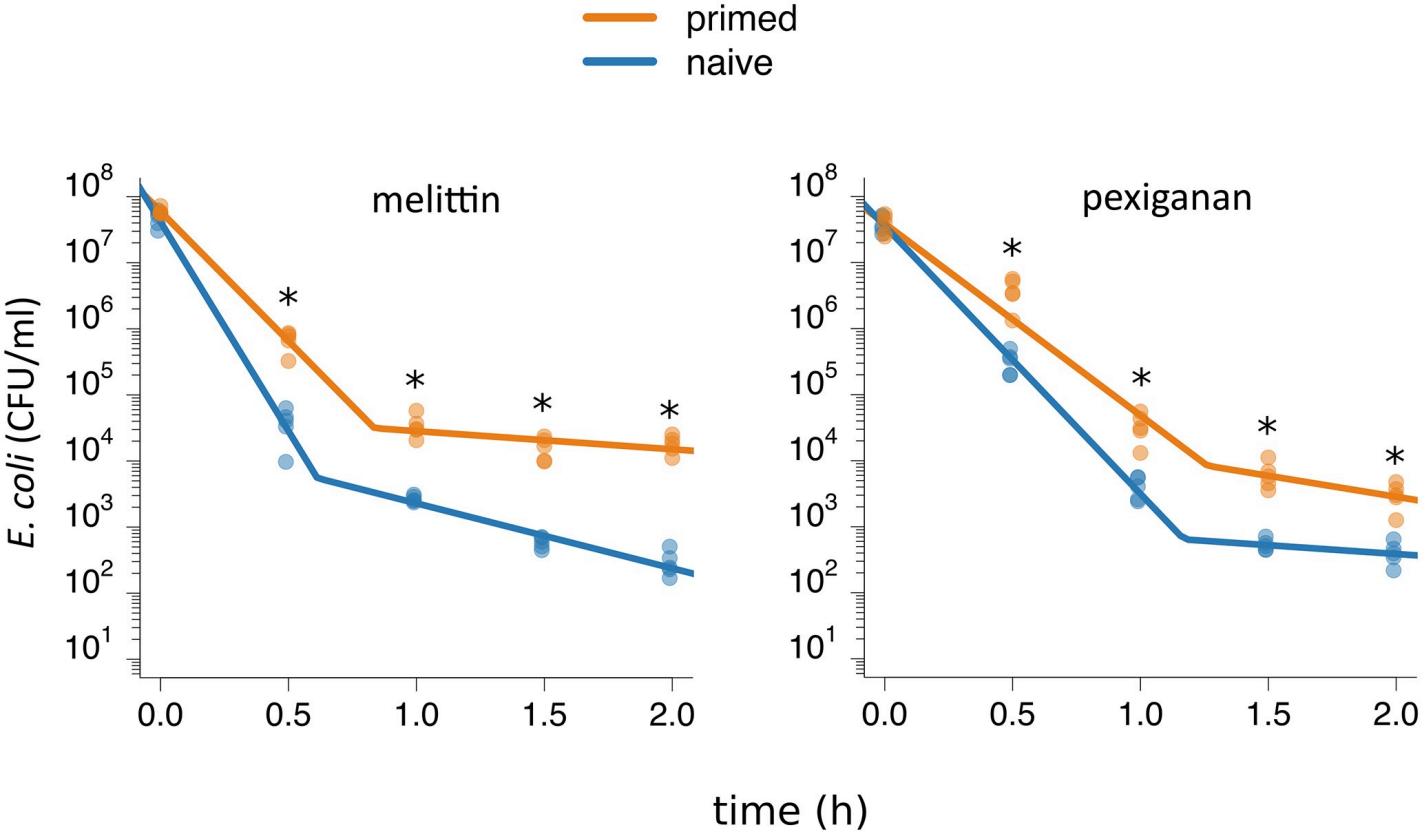
Bacterial tolerance and persistence determine the shape of time-kill curves. Time-kill experiments with *E*. *coli* K-12 MG1655: primed (orange) and naive (blue) bacteria were exposed to 10xMIC of (A) melittin and (B) pexiganan. At each time-point, we measured the bacterial population size 5 times. We tested if priming influenced tolerance and persistence with contrasts following an ANOVA. For both antimicrobials, priming significantly increased the slope of the first phase, the measure of tolerance, and the bacterial level in the second phase, the measure of persistence, (significance level: *p* < 0.05). We corrected for multiple testing with the Bonferroni-method. The line in the plots indicates the best fit of a biphasic function (S2 Table), on which our statistical analysis is based.

We primed *E*. *coli* K-12 bacteria *in vitro* by exposing them for 30 minutes to a decimal fraction of the minimal inhibitory concentration (MIC) of the two AMPs, pexiganan and melittin (MICs were 1 and 2 μg/ml respectively, S1 Table). This concentration was selected because it does not significantly change the bacteria doubling time under the tested conditions. Subsequently, we exposed the primed bacterial populations to lethal concentrations of the respective AMPs (10xMIC, S1 Table) and then monitored bacterial survival over time. We found that the priming treatment resulted in much higher *E*. *coli* survival (Fig 1). To exclude the possibility that increased survival is caused by a mutant subpopulation, we determined the minimal inhibitory concentration (MIC) after priming and after challenge (triggering), including the entire surviving fractions. The MIC remained unchanged for all conditions (S1 Table), showing that the response is purely phenotypic.

### Killing curves show a biphasic shape

The decline of the time kill curves is biphasic, suggesting two subpopulations. This coincides with killing patterns described when persister cells are present [29–32]. We also excluded that deviations from monophasic decline arise because of decreasing antimicrobial concentrations over time (S2 Fig): we took the supernatant containing either of the AMPs (after centrifugation and sterilization by filtration) from a killing experiment. We then used this to re-suspend a similar amount of bacteria. The resulting killing showed no statistical differences to the first experiment.

We fitted the time-kill curves to a biphasic linear function. For both AMPs, bacterial populations declined faster during the first compared to the second phase (Fig 1, S2 and S3 Tables). Tolerance, the decline of bacterial populations in the first phase, was significantly higher in primed than in naive bacteria for both AMPs. Priming also resulted in a higher number of persisters, as depicted by higher survival in the second phase. The change in population size in the second phase, however, was not significantly different between primed and naive populations, indicating that the population dynamics as such in the second phase are not influenced by priming (Fig 1). In short, priming with AMPs allow bacteria to survive better by increasing both bacterial tolerance and persistence.

**Fig 2:**
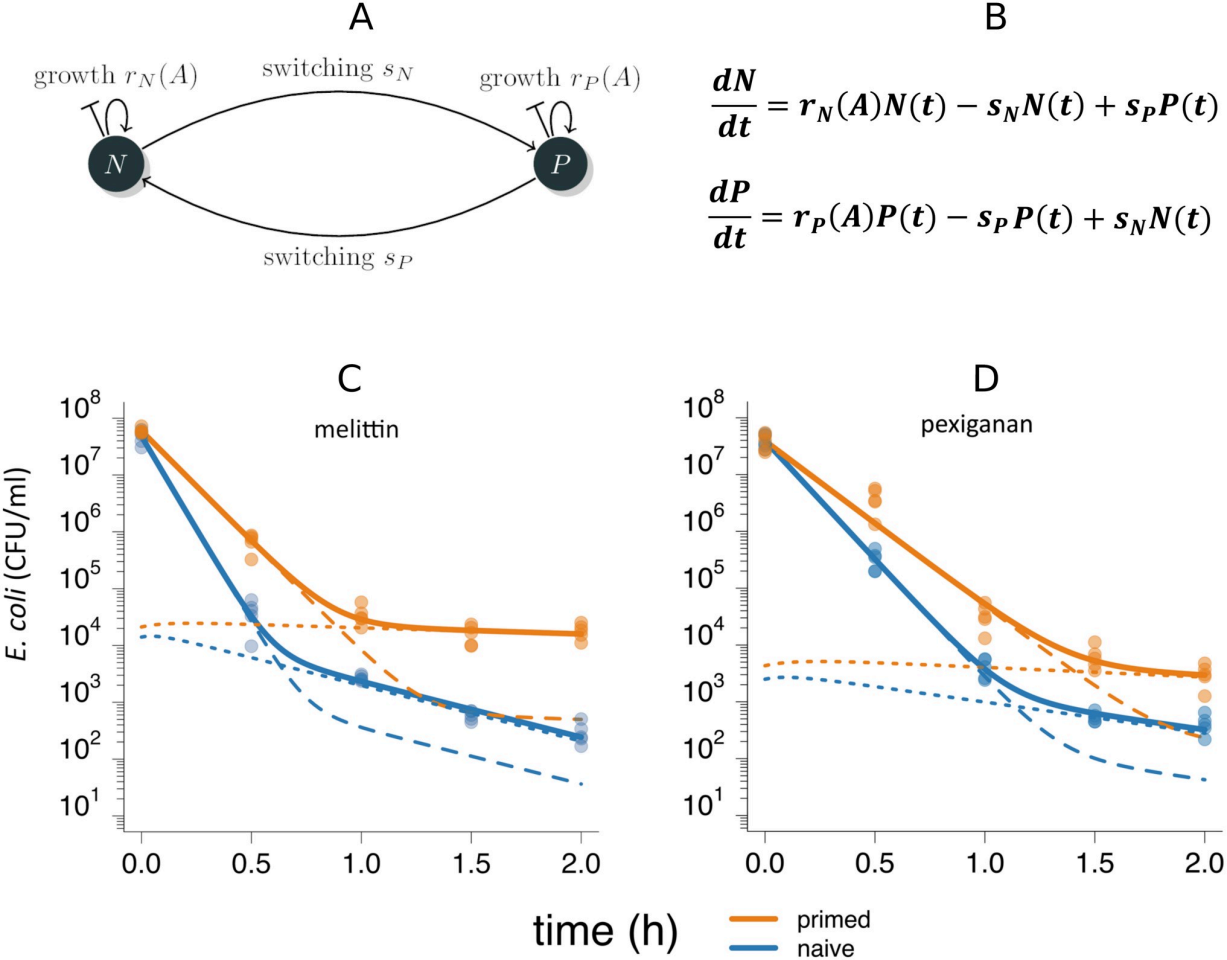
The two-state model describes time-kill data. (A) The two-state model (adapted from *29*) consists of two subpopulations, normal cells (*N*) and persister cells (*P*) and is parameterized with the growth rates *r*_*N*_(A) and *r*_*P*_(A), which are dependent on the concentration of AMPs (A) in the system, and the switching rates *s*_*N*_, and *s*_*P*_. Each subpopulation is described with an ordinary differential equation (B), which describes the change of the respective subpopulation over time. For each antimicrobial, melittin (C) and pexiganan (D), we fitted the model to the data of naive (blue) and primed (orange) bacterial populations (see also Fig 1) individually. The continuous lines represent the total bacterial population *B(t)*, with *B(t)* = *N(t)* + *P(t)*, and the dashed and dotted lines represent the subpopulations *N(t)* and *P(t)*, respectively. Bacteria primed with melittin have an increased net growth rate *r*_*N*_ and decreased *s*_*P*_ compared to the naive populations. In the case of pexiganan, the parameter *r*_*N*_ is significantly higher in primed compared to naive populations. For an overview of the fitted parameters, see S5 Table.

To quantitatively assess the relationship between the above observations and the population dynamics of the bacteria, we fitted a mathematical model developed by Balaban *et al*. [29] (S3A Fig and S3 Table) to the time kill data. This model assumes that bacteria exist in two phenotypic states, normal cells (*N*) and persisters (*P*). The two subpopulations *N* and *P* differ in their susceptibility to AMPs, a difference that is implemented as differing net growth rates for a given amount of AMPs (A), with *r*_*N*_*(A)* and *r*_*P*_*(A)*, respectively. Bacteria switch from subpopulation *N* to *P* with the rate *s*_*N*_ and back with the rate *s*_*P*_. Fitting this model revealed that priming affected two of the three estimated bacterial traits (S6 Fig): the net growth rate (*r*_*N*_) of the non-persister subpopulation increased, which translates into increase in tolerance, and the switching rate (*s*_*P*_) of persistent cells back to a growing and therefore susceptible state decreased. Together, these effects result in higher persistence levels in our model (Fig 2 and S13 Fig).

### On-chip fluorescent microscopy shows differential killing induced priming response

**Fig 3:**
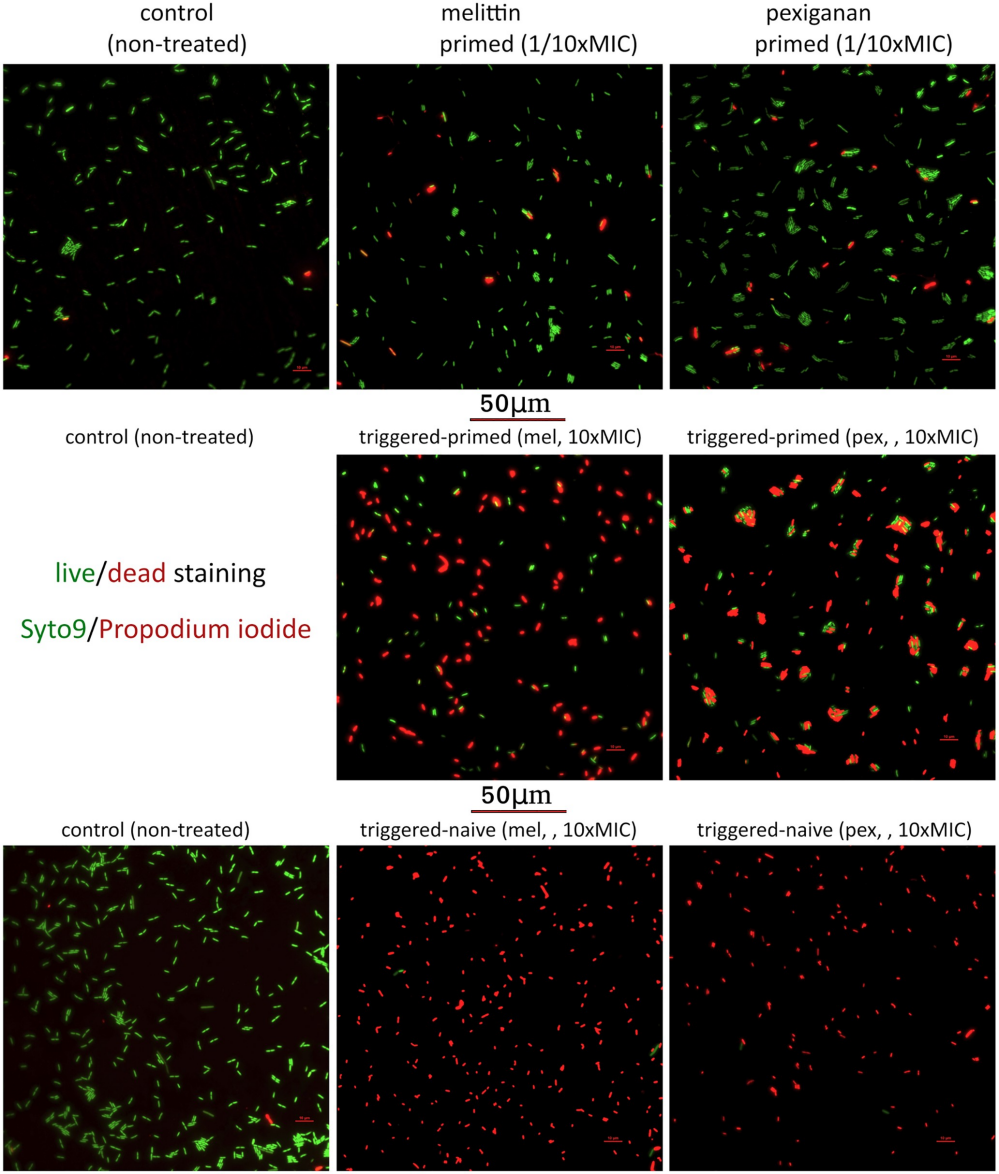
Cell viability after treating with priming and trigger doses of melittin and pexiganan were determined using the live/dead BacLight Bacterial Viability Kit (Thermo Scientific, Germany) on-chip as described in the Material and methods section. After priming during 30 minutes, the treatments were removed by perfusing fresh MHB. The cells were allowed to recover for another 30 minutes and challenged with 10xMIC for 10 minutes. The AMPs were quickly removed by perfusing fresh MHB supplemented with syto9 and propidium iodide. The fluorescence images were acquired as described in the Material and methods section.

After lethal exposure to melittin and pexiganan we observed a high degree of heterogeneity regarding the killing of individual primed cells, with many cells surviving the killing concentrations inside an *ad hoc* microfluidic device (Fig 3). This device ensures to keep all the cells in focus, small consumption of AMPs and fast removal of treatment and staining (S4 Fig). Almost all of the non-primed bacteria were killed after a short time of exposure. As melittin and pexiganan both damage the membrane, which ultimately leads to cell death, the live-dead stain—despite its limitations [33]—seems to be suitable here for visualization purposes. In addition, we used colony forming unit (CFU) counts to estimate killing. Primed cells also aggregate, with a stronger effect in pexiganan-treated cells compared to melittin-treated cells (Fig 3). Aggregation is also consistent with the production of some extracellular components such as colonic acid production or curli (see below) [34].

### Priming is mainly mediated by either curli or colanic acid biosynthesis pathway activation

**Fig 4:**
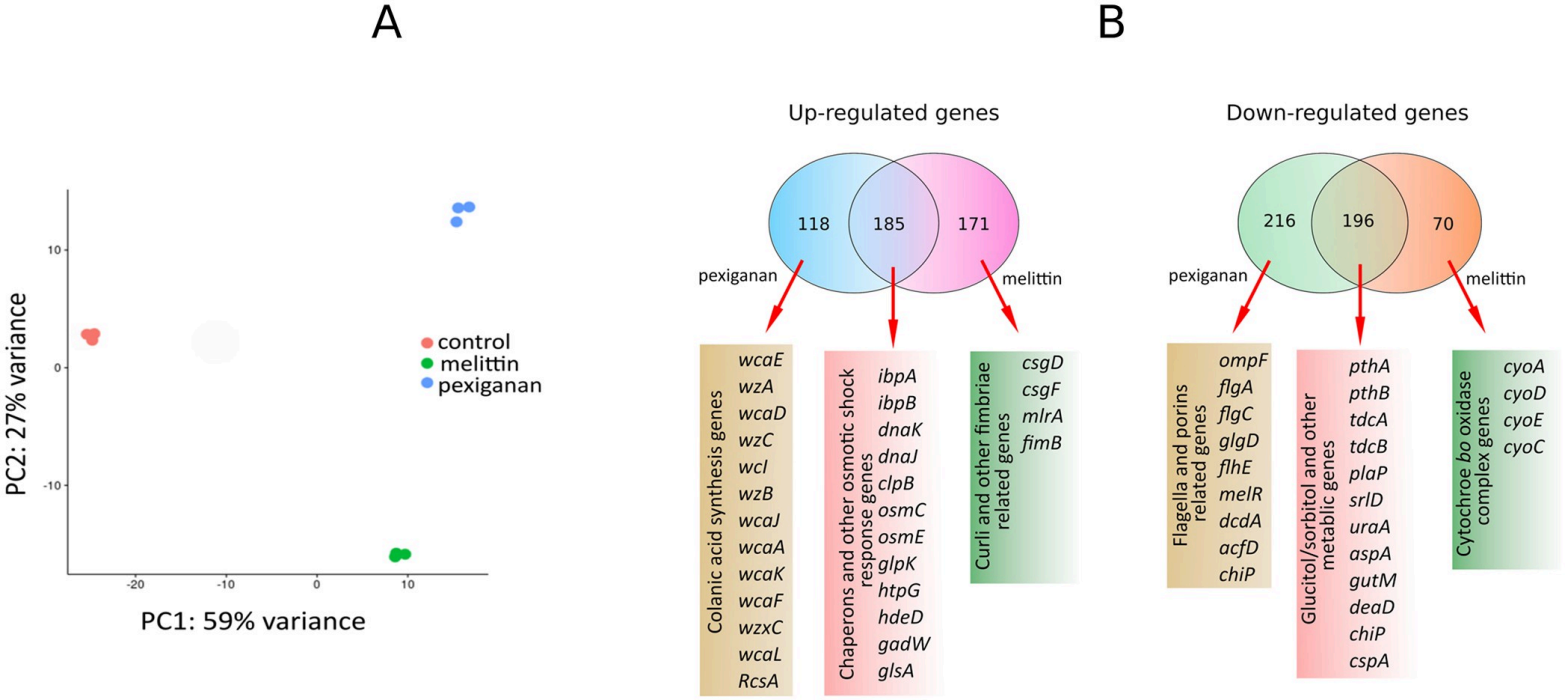
Gene expression in primed *E*. *coli*. (A) Principal component 1 separates the control from the peptide priming, PC2 separates the melittin induced response from the pexiganan response. (B) Venn diagrams showing specific and overlapping responses of *E*. *coli* MG1655 to priming concentrations of melittin and pexiganan.

**Fig 5:**
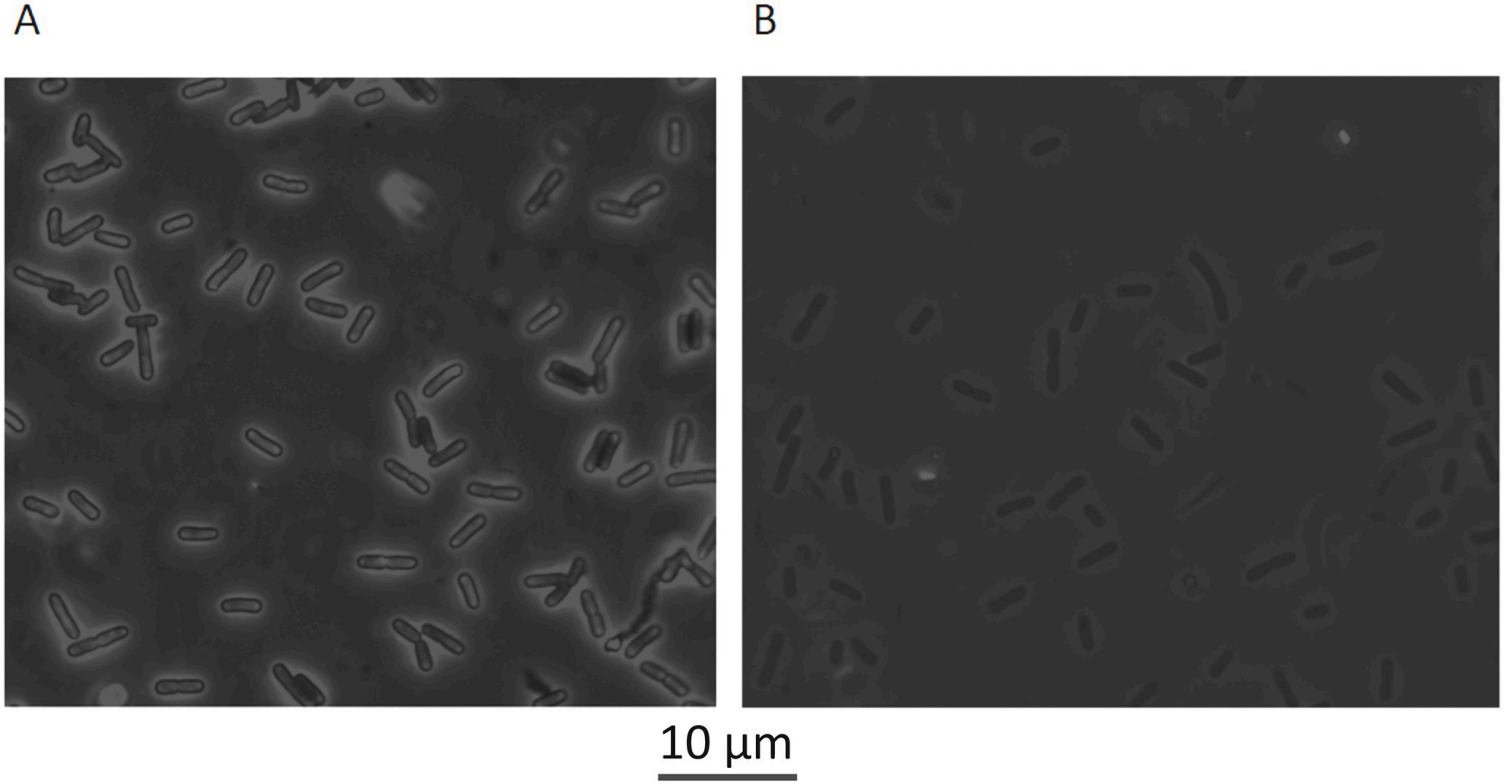
*E*. *coli* MG1655 treated for 30 minutes with 1/10xMIC (priming concentration) of pexiganan (A) and non-treated bacteria (B, control) observed under phase contrast microscopy. The specimens consisted of cells suspended in a 0.1% solution of nigrosin to create a strong contract to visualize the colanic acid capsules. Bacteria were observed with 1000X magnification.

**Fig 6:**
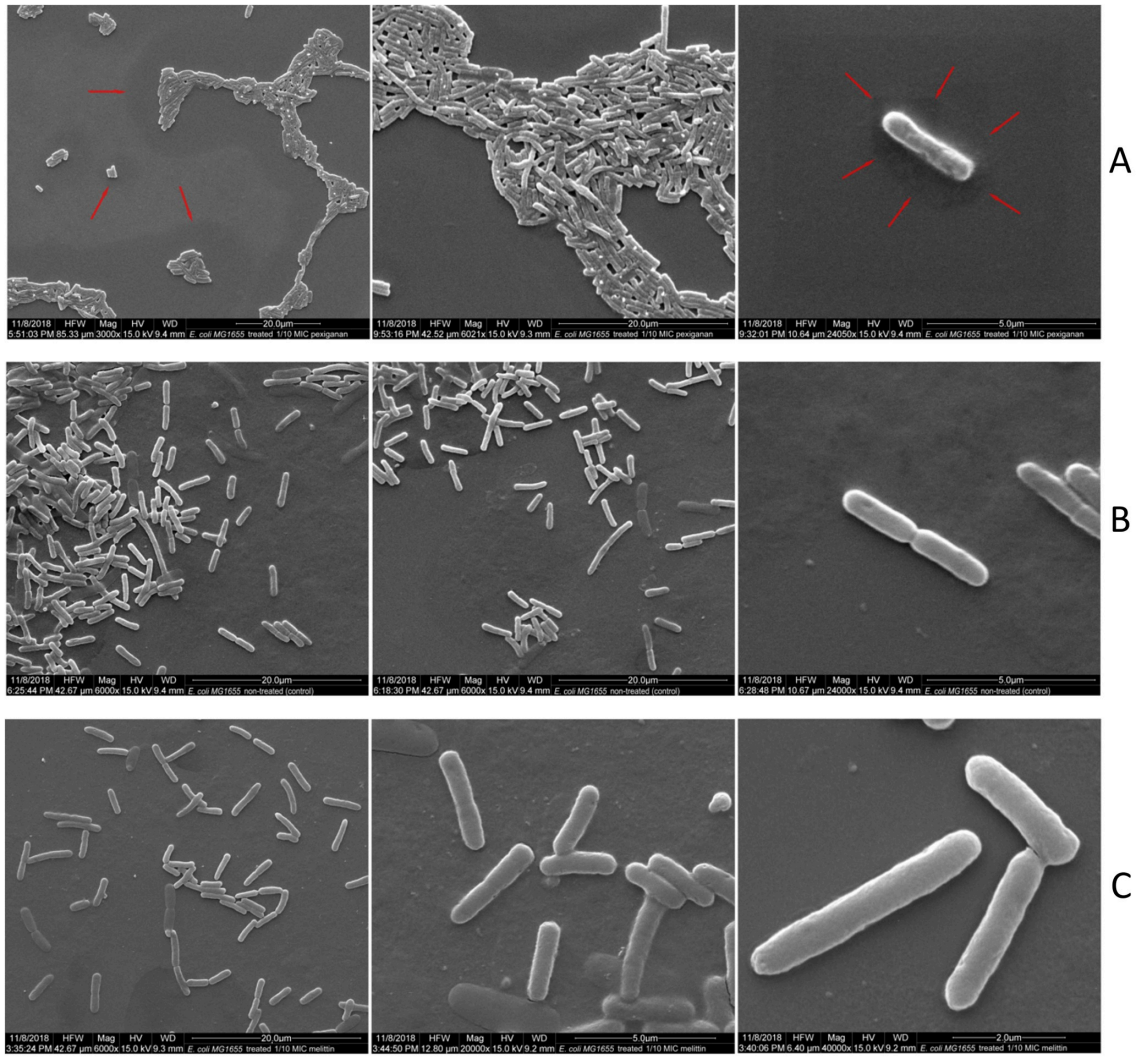
SEM of *E*. *coli* MG1655 treated with 1/10xMIC (priming concentration) of pexiganan (A) and melittin (B) and non-treated bacteria (B, middle row, control). Bacteria were treated with the AMPs for 30 minutes before sample fixation. No apparent differences were noticed between melittin-treated cells (C) and controls. In case of pexiganan, the treated cells tend to aggregate, a phenotype that is consistent with the presence of colanic acid. Red arrows indicate shadowed areas potentially produced by the capsule of colanic acid that collapse fixation and dehydration. Bacteria were observed with different magnifications ranging from 3000X to 40000X.

To understand how priming leads to tolerance and persistence, we used RNAseq of cells exposed to priming concentrations of AMPs (Fig 4, see full dataset in S4 Table). A principal component analysis shows clear segregation by treatment among the control and pexiganan or melittin treated bacterial groups (Fig 4A). Additional analysis, such as correlation among the biological replicates, indicated good quality data for the RNA profiling experiment (S5 Fig). Exposure to sub-lethal concentrations of pexiganan (0.1xMIC, as above) induced colanic acid synthesis (Fig 4B). This was confirmed by phase contrast imaging. We observed the formation of a characteristic colanic acid capsule in pexiganan-primed but not in naive cells (Fig 5). The priming response was homogeneous, with all observed cells producing a colanic acid capsule under priming conditions. Colanic acid capsules have been shown to protect against AMPs and antibiotics [35–37]. The presence of colanic acid could also be observed via scanning electro-microscopy (Fig 6).

**Fig 7:**
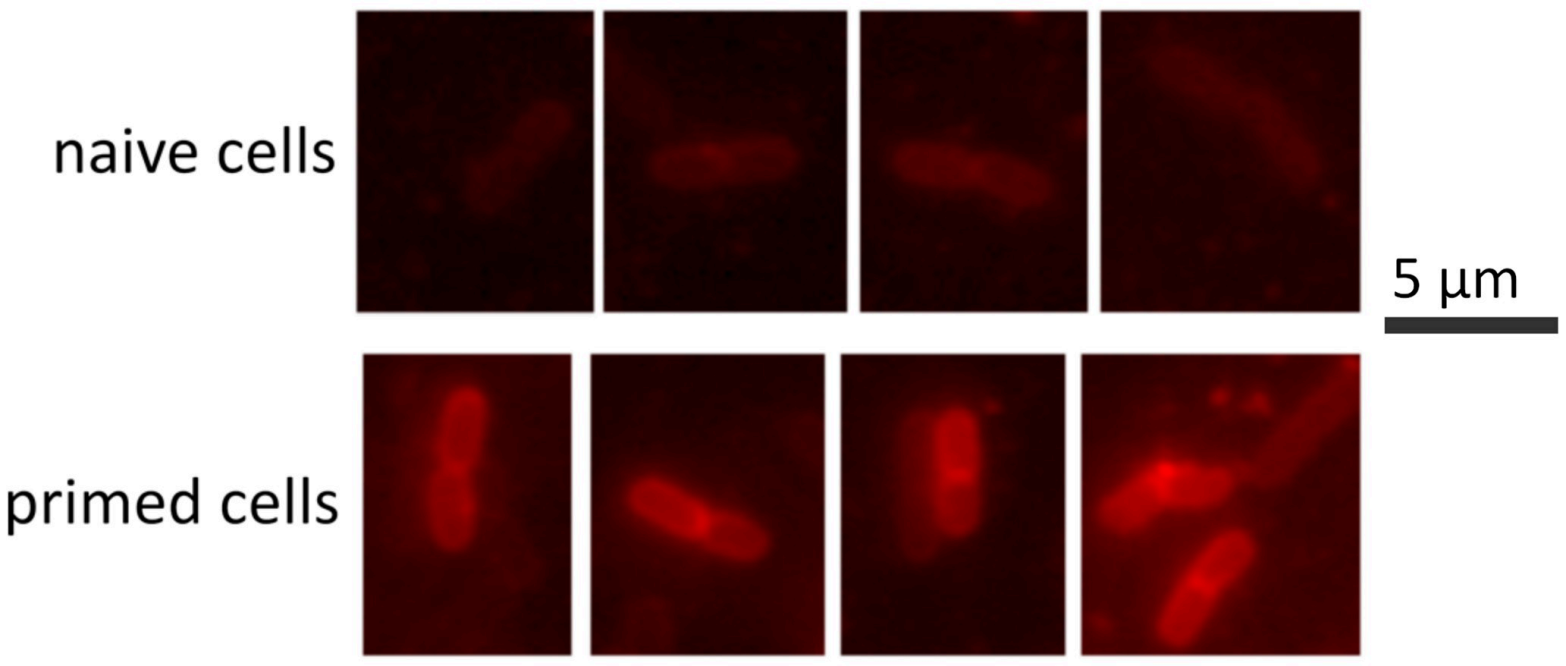
Detection of curli production by primed cells after exposure to a killing dose (10xMIC) of melittin for 30 minutes before image acquisition. Curli production is only detected in a small proportion of primed cells (survival fraction). After treatment, we exposed the cells to ECtracer 680 (Ebba Biotech, Sweden), a red fluorescent tracer molecule for staining of curli.

Exposure to a sub-lethal concentration of melittin (0.1xMIC, as above) induced up-regulation of curli fimbriae (Fig 4B). We could also detect the presence of curli on the bacterial surface of melittin-treated bacteria. The addition of a red fluorescent chemical, ECtracer 680, that specifically binds to curli fibres strongly stained the primed cells but very poorly the naive ones (Fig 7). Curli is an important virulence factor and a component of the extra-cellular matrix that protects against AMPs and enhances bacterial survival in an in vivo model [38]. In contrast to colanic acid, we failed to visualise curli fimbriae by scanning electro-microscopy, probably because curli fibres were too short.

**Fig 8:**
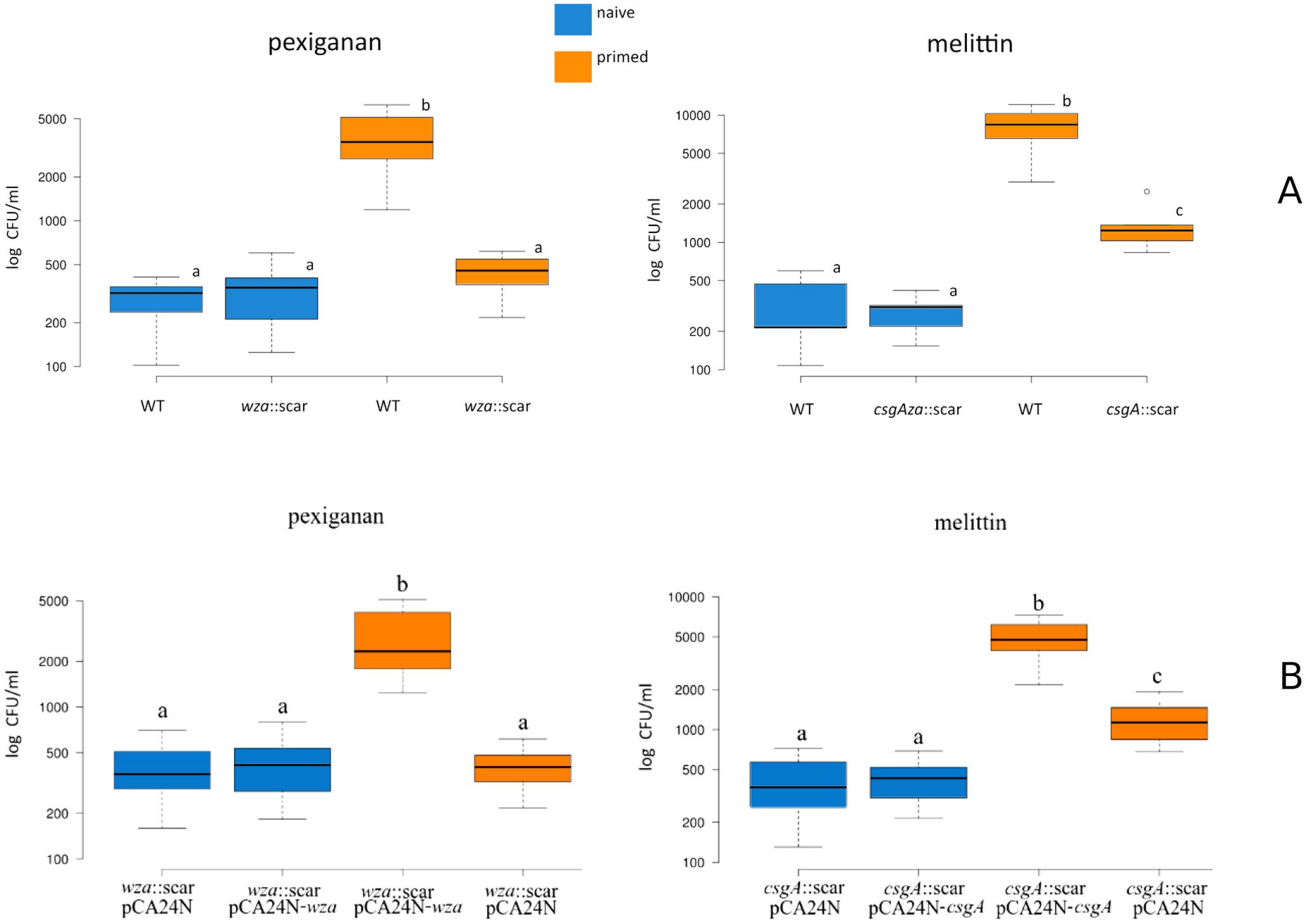
Boxplots data show deficient priming responses of the *E*. *coli* MG1655 mutants *wza*::*scar* and *csgA*::*scar* when they are exposed to triggering concentrations of pexiganan and melittin respectively (top panel). Complementation restores the lost priming capacity for both mutants in *E*. *coli* MG1655 *csgA*::*scar* and *wza*::scar (down panel). The strains were complemented with the plasmids pCA24N*-*wza and pCA24N*-csgA* from the ASKA collection. Note that control cells that were transformed with the cloning vector pCA24N show a decreased priming response similar to the one from the mutants. The statistical differences were tested by one-way ANOVA and Dennett’s tests. Different letters highlight significant differences (*p*<0.05).

The removal of an essential gene for colanic acid production (*wza*) completely abolished the priming effect of pexiganan (Fig 8). A curli-deficient mutant (by *csgA inactivation*) showed a significant decrease in the priming effect induced by melittin (Fig 8). Both AMPs also induced significant overlap in gene expression related to osmotic shock (Fig 4). The introduction of the plasmids *pCA24N-csgA and pCA24N-wza* recovered the priming response in contrast to the cloning vector (pCA24N) that did not. This indicates that the observed phenotype (priming deficiencies) in the mutants (*csgA*::Kn for curli and *wza*::Kn for colanic acid) can be attributed to these two pathways, colanic acid and curli synthesis (Fig 8).

### Priming and persistence

**Fig 9:**
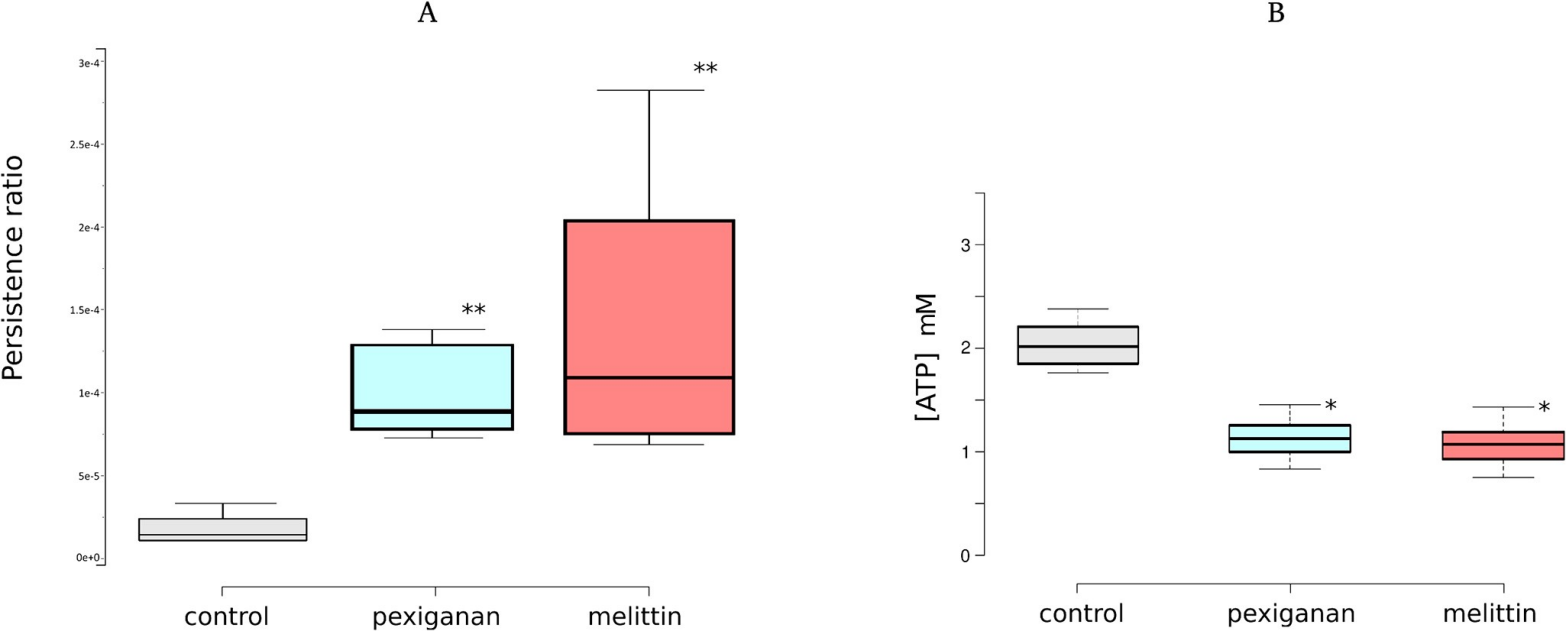
Cell viability and persister formation after AMP priming. The number of persister cells was determined after treating with priming doses of melittin and pexiganan. After priming for 30 minutes, the treatment was removed by centrifuging and washing cells twice with 2 ml of fresh MHB. Ciprofloxacin was added to a final concentration of 2 μg/ml and survival was determined by plating in MHB agar after 4 hours’ incubation (panel A). Before the addition of ciprofloxacin, the intracellular ATP concentration was also measured (panel B). The pre-treatment with melittin or pexiganan did not change the MIC of *E*. *coli* to melittin, pexiganan or ciprofloxacin (S1 Table).

To determine if priming stimulates persistence, we used an assay based on the killing of fast growing bacteria under a treatment with the antibiotic ciprofloxacin, where only persister cells survive [39]. We found that the treatment of *E*. *coli* with priming concentration of melittin and pexiganan significantly increased the number of persisters cells (Fig 9A). We also checked that the pretreatment with melittin or pexiganan (priming) does not change the minimal inhibitory concentration to ciprofloxacin (S1 Table).

It has been also shown that lower ATP levels resulted in decreased antibiotic target activity thereby leading to persister formation. Lower ATP levels could induce the arrest of cell division by the action of toxin-antitoxin systems [39]. Because persisters are cells that by definition are metabolically inactive, the dormant state could be induced or correlated with low concentrations of ATP [40]. As ATP leakage is a hallmark of AMP-treated bacteria [41], we hypothesized that exposure of bacteria to the pore-forming AMPs melittin and pexiganan would lead to ATP leakage by changing membrane permeability. The level of intracellular ATP in the AMPs-treated cultures was significantly lower in primed bacteria for both AMPs as compared to controls, which could contribute to the significant increase in persisters (Fig 9B). The pre-treatment with melittin or pexiganan did not change the MIC of *E*. *coli* to melittin, pexiganan or ciprofloxacin, consistent with the definition of persisters [16].

### The priming response of melittin or pexiganan does not result in cross-protection

We did not find any significant cross-protection when bacteria were primed with pexiganan and challenged with melittin or *vice versa* (S7 Fig). These results suggest that the bacterial response shows some degree of specificity and that the specific molecular targets of melittin and pexiganan may differ.

### A mathematical model predicts role of persistence and tolerance in resistance evolution of primed cells

To model resistance evolution using population dynamical models requires to quantify the influence of priming on tolerance and persistence, phenomena inherently linked to the growth dynamics and subpopulation structure. We extended the two-state population model of Balaban *et al*. [29] by a resistant population (S3 Fig). For these simulations, we used the parameter estimates that we obtained by fitting the model to time-kill datasets of melittin and pexiganan (S10 Fig and S5 Table) and assumed a zero-order pharmacokinetic profile (S9 Fig). In our simulations, we investigated the effect of priming on the time to clearance and the probability of resistance evolution, similar to previous work [9]. Our simulation approach allowed us to investigate the effects of priming and persistence on treatment failure in isolation and combination, which is experimentally out of reach.

**Fig 10:**
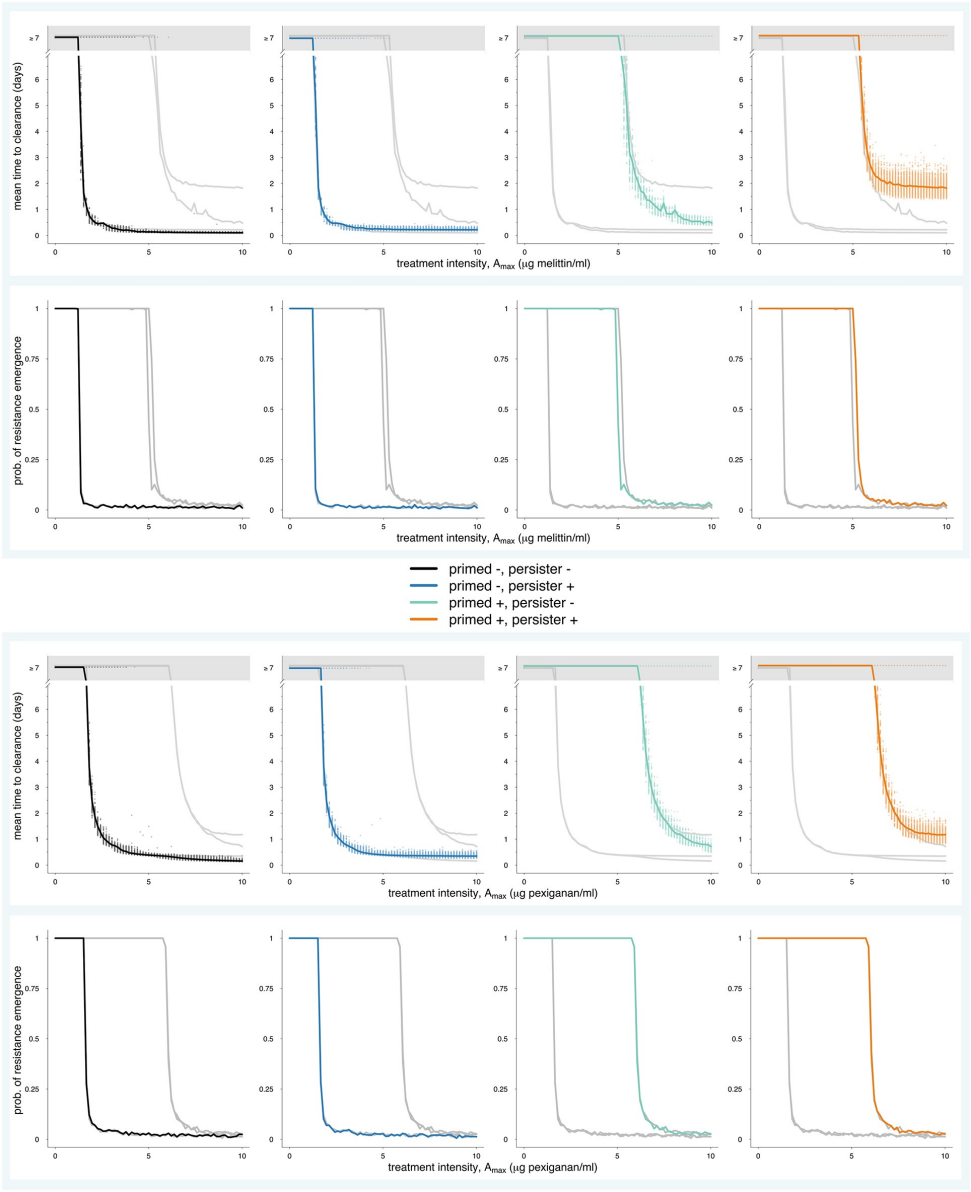
Influence of priming on time until clearance and resistance evolution. We extended a previously developed pharmacokinetics (PK) and pharmacodynamic (PD) framework to include persistence (S3B Fig). With this framework, we estimated PD curves (S7 Fig) for primed and naive bacteria and the estimated switching rates (Fig 2) to predict time until clearance and probability of resistance evolution. We simulated primed bacteria with heterogeneous population consisting of *N* and *P* subpopulations (primed +, persisters +), and naive bacteria with heterogeneous subpopulation (primed -, persisters +). In addition, we simulated dynamics without persistence for both primed and naive bacteria (primed +, persisters - and primed -, persisters–). In in row 1 and 3, each dot is an individual run. The lines denote the average of the respective runs. No clearance (grey area) means that simulated treatment could not reduce bacteria population < 1 cell within 7 days of treatment. For comparison, we plotted all simulation results in each plot. All parameter values used in the mathematical model are listed in S5 Table.

We found that priming translates into an increased time until clearance for intermediate and high treatment intensities, i.e. doses of AMP. We found that survival of the population was highly dependent on priming (Fig 10), however, the presence of persistent cells alone only marginally increased time until clearance at high AMP concentrations. This means that the main increase in survival of bacterial population is due to tolerance. While for naive populations (primed -), persistence does not prolong mean time to clearance, primed populations benefit of having a persistent subpopulation at high treatment intensities. Here, not only is the mean survival increased, but also the variability between individual runs is higher, making future outcomes less predictable. The results do not qualitatively change for larger pharmacokinetic decay rates (*k*), typical for AMPs [1] (S12 Fig). Taken together, an increase in tolerance alone resulted in higher survival independent of the presence of persistence. An increase in persister cells further increased survival at high antimicrobial concentrations.

Next, we assessed how priming affects resistance emergence. Generally, bacterial resistance evolution depends on the population size, mutation rate and the replication rate. Although exposure to antimicrobials such as AMPs can increase resistance evolution [42], we have previously shown that AMPs do not increase the mutation or recombination rates [43,44]. Hence, while mutation rate remains constant, priming increases bacterial survival and therefore population size, it also increases the number of persisters that do not replicate and are a very limited source of resistant mutations (Fig 10) and that the beneficial effects of priming on survival due to increased persistence did not translate into an increased probability of resistance emergence and establishment (Fig 10). The probability of resistance emergence was mainly influenced by the effect of priming on tolerance.

## Discussion

We find that sub-lethal doses of the AMPs melittin and pexiganan prime bacterial cells to increase both tolerance and persistence. As sub-lethal concentrations of antimicrobials (AMPs and antibiotics) are common in the environment and in hosts/patients [19,45], a priming response, if it is a widespread phenomenon, would have significant consequences. Antimicrobials that prime, would inevitably induce the formation of tolerant and persisting cells. This should apply to situations of induced immune responses, but equally to drug treatments. In short, generating or increasing populations of tolerant and/or persistent bacteria, would be inevitable in many situations, and would represent a serious obstacle to clearing bacterial infections [17].

Our study is limited to the effect of single antimicrobial peptides. While in many natural situations cocktails of AMPs are expressed [1] there are other situations where individual AMPs dominate. One example is given by the cnidarian *Hydra*, that uses NDA-1, a peptide with a dual function as antimicrobial and neuropeptide to shape its microbiota [46]. Some antimicrobial peptides and proteins (e.g. lysozyme) are constitutively present, and synergistic action with low dose of additional AMPs may still result in active concentration levels. Finally, if certain bacteria inhibit intrinsic AMP-resistance, a situation where only one component of a defence cocktail is active is possible. Some *Staphylococcus aureus* virulence factors inhibit complement activation [47] and only a few human defensins are effective, abolishing or limiting the cocktail effect [48]. Besides, the activity of other active molecules such as defensins is limited to some tissues or organs [49].

The priming response we report is mediated by rather general phenotypic resistance mechanisms. The molecular basis of the induction of AMP tolerance and persistence rely on modifications of bacterial envelopes involving either curli production under melittin treatment, or colanic acid production after pexiganan exposure. Curli is the major proteinaceous component of a complex extra-cellular matrix produced by many *Enterobacteriaceae* and is involved in many physiological and pathogenic processes. Curli fibres are involved in adhesion to surfaces, cell aggregation, and biofilm formation, including the adhesion phase. Curli can also mediate host cell adhesion and invasion and they are potent inducers of the host inflammatory response [50]. It is known that curli fibres protect uropathogenic *E*. *coli* against the human antimicrobial peptide LL-37 [51]. Interestingly, LL-37 also inhibits the polymerization of curli fibres by direct interaction, which indicates a competition between polymerization that confers resistance, and inhibition of polymerization that would preserve sensitivity [51].

Colanic acid is an exopolysaccharide secreted by *E*. *coli* and a number of other *Enterobacteriaceae* assumed to promote biofilm formation and to protect the organism when exposed to harsh environments [52,53]. The exopolysaccharide colanic acid is regulated by the two-component Rcs phosphorelay and has traditionally been associated with biofilm formation and protection from desiccation [54]. Other studies have shown that colanic acid also contributes to serum survival [55–57]. It is important to note that the bactericidal component of mammal serum is based on cationic peptides such as those in the membrane-attack complex of complement [58] and antimicrobial peptides. A transcriptomic response to human serum revealed increased gene expression of the colanic acid biosynthesis operon [57] and colanic acid was protective against the bactericidal effects of human serum [57].

Interestingly, the activation of both pathways shows different dynamics in biofilm formation [59]. Curli and colanic acid are important components of the biofilm matrix, curli for the primary adhesion and colanic acid for the biofilm structure [50,60]. Triggering their expression by sublethal levels of AMPs, could potentially catalyse biofilm formation. Within a host, if the immune system fails to clear the pathogens, the AMP-priming effect may thereby favour the transition from acute to chronic bacterial infections, where biofilms prevail. Both curli and colanic acid protect the cells by shielding them from AMPs making access to the outer membrane more difficult and/or capturing AMPs. While this provides scope for cross-protection, we did not find evidence for cross-protection.

In natural systems of host-microbe interactions, priming plays a role in facilitating colonization of AMP rich environments [11]. Priming by AMPs also plays a role in infection vectors: in the flea gut the PhoQ-PhoP system is induced in *Yersinia pestis*, the infective agent causing plague, by AMPs leading to biofilm formation that enhances transmission to the final host [61]. It is not clear as yet if non-inherited AMP-resistance will facilitate opportunistic infections in a way similar to genetic AMP-resistance, as has been shown for genetic AMP resistance in *S*. *aureus* [62], but in the light of our results it seems likely.

Tolerance and persistence can drive the evolution of genetic resistance against antibiotics [10,20]. We find that, while priming *E*. *coli* with AMPs results in increased tolerance and persistence, the main driver of resistance evolution in our model is increased tolerance. Resistance evolution is a product of mutation supply and strength of selection. The selection in our scenario is strong, but mutations are only supplied from the subpopulation of tolerant cells, not from the persister cells, as they are metabolically inactive. Therefore, while the persisters do not contribute to resistance evolution, they might provide a source for secondary infection, once the immune system overexpression ceases or alternatively, when the antimicrobial drug is removed. In antibiotic resistance evolution, by contrast, persisters have been shown to contribute to resistance evolution [63]. Part of this seems to be explained by increased mutagenesis caused by the antibiotics [63], an aspect we explicitly did not model as AMPs do not increase mutagenesis [43].

To understand the mechanisms of priming we studied two AMPs. This situation is comparable to for example drug testing or in natural settings the presence of single AMPs such as pleurocidin in the mucus of the winter flounder [64]. In many natural situations though, bacteria encounter suites of AMPs [1] (and references therein). Future experiments will need to explore the role of priming in these more complex environments.

Our combined theoretical and empirical results suggest that in hosts the optimal strategy of AMP-deployment would be a fast increase in concentration to avoid priming and the subsequent development of non-inherited resistance through both increased persistence and increased tolerance. Such fast increases in AMP concentrations in specific locations are realised in some important natural situations. During insect metamorphosis, when the gut is renewed in the pupa, a cocktail of AMPs and lysozymes is discharged into the gut ([65] and references therein), resulting in a quick reduction of bacterial numbers by orders of magnitude. When infections persist, one possible solution is a long-lasting immune response [66] that deals with a recurrent infection that could potentially be caused by persisting cells. These natural situations also have parallels in the medical application of antimicrobials. We propose that a fast increase of antimicrobial concentration, as for example in the intra-venous application of antibiotics, should also reduce the probability of persister formation via priming. It is noteworthy that AMPs in many situations are present in combination with other peptides and frequently these combinations are synergistic [1,67].

Long-lasting antimicrobial exposures are prevalent in natural systems. This is at odds with the observation that long-lasting drug treatments, at least in the case of single drug applications, select for drug resistance. Therefore, at first glance extended treatments do not seem to be a good strategy to manage persisters.

## Material and methods

### Bacteria and growth conditions

*E*. *coli* MG1655 was used as a bacterial model for all experiments. All cultures for antimicrobial tests were grown in Mueller-Hinton I Broth (Sigma). For genetic manipulations we used *E*. *coli* BW25113. All strains and their derivatives were routinely cultured in Lysogeny Broth (LB medium) or SOB (Super Optimal Broth), supplemented with antibiotics when appropriate. All strains used in this study are listed in S6 Table.

### Minimal inhibitory concentration (MIC)

MICs were determined according to CLSI recommendations by microdilution [68] with minor modifications for antimicrobial peptides [69]. Inoculum size was adjusted to 2×10^7^ CFU/ml from a 2-hour mid-exponential phase obtained by diluting 100 μl of overnight cultures in 10 ml of fresh medium in 50 ml Falcon tubes to be consistent with the downstream experiments. The MIC was read as the antimicrobial concentration that inhibited growth after 24 h of incubation in liquid MHB medium at 37°C. Polypropylene non-binding plates (96 wells, Th. Geyer, Germany) were used for all experiments. MIC was also determined similarly as above for primed and triggered bacteria after two and a half hours’ exposure to priming and triggering concentrations of AMPs. In this case, we used the entire surviving fraction after tenfold dilution in MHB containing 10xMIC of the antimicrobials. The bacteria were incubated for 24 hours. The values were compared with naive controls for both antimicrobial peptides, melittin and pexiganan, and for ciprofloxacin.

### Priming experiments

Bacterial cultures were diluted 1:100 from a 16-hour overnight culture (by adding 100 μl to 10 ml of MHB medium in 50 ml Falcon tubes). Then, the bacteria were grown for 2 h to reach approximately 2 × 10^8^ CFU/ml. Starting from 1x10^8^ CFU/ml (mid exponential growth), 2 ml of bacterial cultures were exposed (stimulus) to 1/10 MIC of melittin or pexiganan for 30 minutes at 37°C with soft shaking. The tubes were centrifuged at 4000 x g for 10 minutes, the supernatants were removed by sterile aspiration and the pellets were resuspended in fresh MHB and allowed to recover for 60 minutes. The cells were challenged (triggering of the priming response) with a concentration equivalent to 10xMIC. The cultures were diluted and plated to determine cell viability. Five biological replicates were generated. Non-treated cells were used as a control and also harvested during mid-exponential growth.

### Activity of melittin and pexiganan from the supernatant of challenged cells

Similar to the priming experiments, 1x10^8^ CFU/ml mid-exponential phase bacterial cultures (five tubes per group) were exposed to 10x MIC (supernatant I) of melittin or pexiganan for 150 minutes at 37°C with soft shaking. The tubes were centrifuged at 4000 x g for 10 minutes, the supernatant was collected and centrifuged again at 20 000 x g for 30 minutes. The new supernatant was filtrated using 0.22 μm sterile filters (Sigma Aldrich, Germany) and used immediately (supernatant II). In parallel, exponentially growing cultures containing 1x10^8^ CFU/ml per tube were centrifuged at 4000 x g for 10 minutes, the supernatants were removed by sterile aspiration and the pellets were re-suspended in equal volumes of the supernatant II and incubated for 150 minutes at 37°C with soft shaking. Samples from each tube were taken from both supernatant I and supernatant II every 30 minutes and diluted and plated to determine cell viability (five replicates per condition). Data points from time-kill experiments from bacteria treated with the supernatant I (first round) and the supernatant II (second round) were compared to determine changes in the activity of the AMPs indicating degradation or adsorption.

### Persister antibiotic survival assay and intracellular ATP determination

Bacteria from 16-hour overnight cultures were inoculated 1:100 in 2.5 ml cultures of fresh MHB (in 10 ml polypropylene tubes) and the cells were grown for 2 h to reach approximately 2 × 10^8^ CFU/ml. The cultures were then treated with priming concentrations (1/10 MIC) of melittin and pexiganan for 30 minutes. Non-treated cultures were used as controls. All cultures were washed with 2 ml of fresh MHB and centrifuged twice to remove the AMPs. Bacteria were resuspended in equal volumes of fresh medium and 1 ml from each culture was taken to determine the number of persister bacteria at t = 0 by dilution plating on MHB agar. Ciprofloxacin was added to a final concentration of 2 μg/ml to treated tubes and to non-treated controls. The cultures were incubated for four hours. Thereafter, bacteria were washed twice with NaCl 0.9% and plated on MHB agar. The percent survival was calculated as the ratio of CFU/ml before and after the treatment as described previously (final CFU/CFU at 0 h) × 100) [39]. The results are presented as the average from 5 independent biological replications. The remaining 1 ml culture was used to determine the intracellular ATP concentration using a Molecular Probes ATP Determination Kit (Thermo Fisher Scientific, Germany). The cultures were added 9 ml (a ten-time dilution) of lysis solution (Tris-HCl 50 mM, EDTA 5 mM and lysozyme 5 mg/ml) and incubated for 5 minutes for a full lysis. The quality of the lysis was estimated by plating the lysates. Five microliters of lysate culture were used to determine the ATP concentration. The ATP concentration was determined in triplicate.

### Construction and verification of deletion mutants

We inactivated the major curli subunit protein gene *csgA* and the colanic acid precursor gene *wza*. Although both pathways involve many genes, the removal of these two components impair the production of both substances respectively. These mutants were generated in *E*. *coli* K-12 strain MG1655 following the methodology described elsewhere [70]. Briefly, we extracted genomic DNA from the mutants *csgA*::*Kn* and *wza*::*Kn* of the Keio collection [71] (in *E*. *coli* BW25113) and amplified the flanking regions of the kanamycin resistance cassette disrupting both genes and including an appropriate homology sequence. For the *csgA* mutant we used the primers 5’-GATGCCAGTATTTCGCAAGGTG-3’ and 5’-GGTTATCTGACTGGAAAGTGCC-3’ while primers 5’-TAGCGTGTCTGGATGCCTG-3’ and 5’-CCACTTTCAGCTCCGGGT-3’ were used for *wza*. The PCR products were purified, and electroporated into *E*. *coli* MG1655 carrying a red recombinase helper plasmid, pKD46. The strain was grown in 10 ml SOB medium with ampicillin (100 μg/ml) and L-arabinose at 30°C to an OD_600_ of ~0.5. Bacteria were made electrocompetent by washing the cells and centrifuging at 3 000 x g and 4°C for 10 minutes with a cold solution of glycerol 10%. Competent cells in 60 μl aliquots were electroporated with 200 ng of PCR product. Cells were added immediately to 0.9 ml of SOC, incubated for 2 h at 37°C, and spread onto LB agar with kanamycin (30 μg/ml) in 100-μl aliquots. The correct inactivation of genes was verified by PCR. The kanamycin resistant cassette (Kn) was removed for both mutants using the flippase plasmid pCP20.

### Complementation of curli and colanic acid deficient mutants

The constructed strains *csgA*::*Kn* and *wza::Kn were complemented with the respective plasmid pCA24N-csgA and pCA24N-wza from the ASKA collection [72]. The plasmids were introduced into E. coli MG1655 csgA::Kn* and *E. coli MG1655 wza::Kn by electroporation following standard procedures [73]*. The strains were grown in 10 ml SOB medium with kanamycin (15 μg/ml) at 37°C to an OD_600_ of ~0.5 and then made electrocompetent by washing and centrifuging (3 000 x g at 4°C) in a cold solution of glycerol 10%. Competent cells in 80-μl aliquots were electroporated with 1 ng of the purified plasmids. Cells were added immediately 0.9 ml of SOC, incubated during 2 h at 37°C and serially diluted. Aliquots of 100 μl from dilution were spread onto LB agar plates *containing chloramphenicol (30* μg/ml*)* to select for the *transformed cells*. *The strains were also transformed with the cloning vector pCA24N*, *that was used as a control for the complementation experiments*. *Five independent colonies were picked and cultured over-night and glycerol stocks were prepared*. *From these clones*, *mid-exponential phase cells (obtained as described for the previous priming experiments) were grown in 2 ml of MHB supplemented with isopropyl β-D-1-thiogalactopyranoside (IPTG) 100* μ*M*. *The cultures containing 2* × 10^8^ CFU in 10 ml polypropylene tubes, *were primed with melittin and pexiganan as described for the other priming experiment (see above) and challenged with either peptide 60 minutes after a recovery period upon the removal of the priming treatment*. *The tubes were incubated at* 37°C with soft shaking for another 60 minutes and *plated to determine the survival*.

### Transcriptome sequencing

The transcriptome sequencing of primed cells was determined on samples prepared in the same way as described above for the priming experiments. Total RNA from 10^8^ cell per sample was isolated using the RNAeasy Isolation kit (Qiagen, Germany). Traces of genomic DNA were removed from 10 μg of RNA by digestion in a total volume of 500 μl containing 20 units of TURBO DNase, incubated for 30 minutes at 37°C, immediately followed by RNeasy (Qiagen) clean-up and elution in 30 μl of RNase-free water. Following DNase treatment, RNA integrity was assessed using Agilent RNA 6000 Nano kit and 2100 Bioanalyzer instrument (both from Agilent Technologies). Total RNA was depleted from ribosomal RNA using the Ribo-Zero Depletion Kit for Gram-negative bacteria (Illumina, USA). Libraries were prepared using a TruSeq Stranded Total RNA library preparation kit (Illumina, USA) and were sequenced on a MiSeq platform.

Transcript abundances were derived from pseudo-alignment of reads to the cDNA sequences from the ASM584v2 assembly of *Escherichia coli* MG1655 (ENA accession GCA_000005845.2) using Salmon version 0.7.2 with default parameters [74]. Differential gene expression was analyzed using the R package DESeq2 [75] in conjunction with tximport [76]. Pairwise contrasts were performed between the control and each AMP treatment with empirical bayesian shrinkage of both dispersion parameters and fold-change estimation. We defined genes as being significantly differentially expressed when the absolute fold-change in expression was greater than 2, at an FDR-adjusted *p*-value of less than 0.05. The variance-stabilizing transformation was used to remove the dependence of the variance on the mean and to transform data to the log2 scale prior to ordination using principal component analysis. Quality of RNAseq data were contrasted by Euclidian distance and symmetry of data reads distribution (S4 Fig).

### Observation at single cell level

To observe the cell reaction at single cell level during the priming experiments, we used a microfluidic device developed for this project. It consisted of a main channel for bacterial inoculation and medium perfusion and several lateral compartments (1.5 μm height (ensuring all bacteria are kept in focus) and 200 μm width corresponding to the field size of the microscope at 1000x magnification (S5 Fig). The chip was designed in Autocad (version 2018). We started the replication of our microfluidic chips from a custom made (Sigatec SA) silicon (SiO) master. This silicon master was first replicated in Smooth-Cast 310 (Bentley advanced material). Soft lithography was used to produce the chips in PDMS (Sylgard Silicone Elastomer Base and Curing Agent mixed in 10:1 ratio). The PDMS chips were cured overnight at 75°C in an incubator. We punched an inlet and outlet hole for the laminar flow in each chip using a biopsy puncher of 0.5 mm (outer diameter). The chips were bonded to a glass cover slide (24×60 mm) after a 30-second air plasma treatment (PDC-002, Harrick Plasma). Before use, the assembled chip was treated for 15 seconds in air plasma and immediately injected it with filtered MHB medium for passivation. We left the activated chip to incubate for a least 1 hour before loading the bacteria. The devices were loaded to full capacity with a bacterial suspension containing nearly 2x10^8^ CFU/ml (exponentially growing bacteria, OD_600_ 0.5). Cell suspension was injected into the main channel of the chip using a blunted 23G needle attached to a 1 ml syringe. We centrifuged the loaded chip at 200 x g for 10 min using an in-house adapters and checked the loading under a microscope with a magnification of 400x.

After loading the bacterial cells from the outlets of the side channels, we connected the chip to a syringe pump (AL-6000, WPI, Germany) and placed the chip under an inverted microscope. A continuous laminar flow (100μl/h) of MHB through the central channel was maintained throughout the experiment (S5 Fig). For live cell imaging, after infusion with priming or triggering concentration of AMPs, we injected MHB supplemented with bacterial Live/Dead stain kit solutions (Thermo Scientific, Germany, diluted 1:10000, syto9+propidium iodide in MHB) for a final concentration of 0.1 μl/ml of MHB. We took pictures of at least 20 fields per treatment from independent side channels. Fluorescent images were taken of each field of view with simultaneous acquisition in red and green fluorescent channels during a time interval of no more than 2 minutes per treatment with a Nikon Ti-2 inverted microscope (Nikon, Japan). Cells were observed with the 100× objective and controlled by Nis Element AR software. The chip holder is temperature controlled at 37°C.

### Determination of melittin-induced curli

The production of curli was determined by using the fluorescent dye ECtracer 680 (Ebba Biotech, Sweden) that stains extracellular curli. ECtracer 680 was used according to the manufacturer’s instructions. Bacterial cultures were treated on the chip with priming and triggering concentrations of melittin as described above. After priming and triggering, the channels were perfused with MHB supplemented with ECtracer 680 in a proportion of 1/1000 (1 μl of reagent per ml of medium) related to the medium. Cells were observed with the red channel fluorescence for propidium iodide dye using a Nikon Eclipse Ti2 inverted optical microscope (Nikon, Japan) using the 100X oil objective. Two independent samples were prepared for each group (primed and naive cells).

### SEM of *E*. *coli* treated with antimicrobial peptides

Approximately 2×10^7^ CFU/ml *E*. *coli* MG1655 were treated with 1/10 MIC of pexiganan or melittin for 30 minutes respectively. The cultures were concentrated 10 times by a quick centrifugation step of 1 minute at 8000 × g, followed by the extraction of 900 μl of supernatant and resuspension of the remaining 100 μl. After resuspension, 10 μl drops of culture were placed on a circular glass cover slip (1.5 cm of diameter). The drops were fixed with osmium tetroxide vapor for one minute and allowed to dry in a laminar flow cabinet. The cover slips were mounted on aluminum stubs using double-sided adhesive tape and coated with gold in a sputter coater (SCD-040; Balzers, Union, Liechtenstein). The specimens were examined with a FEI Quanta 200 SEM (FEI Co., Hillsboro, OR) operating at an accelerating voltage of 15 kV under high vacuum mode at different magnifications. At least 5 fields from two independent replicates were photographed.

### Statistical analysis

To analyze the priming data, we first tested if the dynamics depicted in the time-kill curves are biphasic.

We fitted the function

$$f(m_{1},m_{2},t_{kink},t)=\{\begin{array}{c} {log}_{10}(CFU(t=0))+m_{1}t,t<t_{kink}\\ {log}_{10}(CFU(t=0))+m_{1}t_{kink}+m_{2}({t-t}_{kink}),t\geq t_{kink} \end{array}$$

to the time-kill data of each AMP and for primed and naive populations individually using a least square algorithm. Here, *t*_*kink*_ is the time point at which the population dynamics switch from the first phase to the second phase and *m*_*1*_ and *m*_*2*_ are the slopes of the first and the second decline, respectively. Note that *m*_*1*_ is a direct measure of tolerance. The standard error (SE) was calculated as

$$SE(\theta )=\sqrt{\frac{1}{I(\theta )}}$$

The parameter *θ* denotes to the estimated parameter values of *m*_*1*_, *m*_*2*_, and *t*_*kink*_ and *I*(*θ*) is the expected Fisher information. The 95% confidence interval was calculated as *θ* ± 1.96*SE(*θ*).

We used an ANOVA by means of contrasts to assess if priming changes tolerance and persistence. For this, we tested if the decrease of the first phase (all data points with t < t_kink_) differed between primed and naive populations (test for differences in tolerance) and if the population size in the second phase (all data points with t > t_kink_) differed between primed and naive populations (test for differences in persistence). Both tests showed significant differences between primed and naive populations for both melittin and pexiganan (significance level: *p* < 0.05). We corrected for multiple testing with the Bonferroni-method.

### Population models

To describe bacterial population dynamics, we used the two-state model by Balaban *et al* [29] (Fig 2A):

$$\frac{dN(t)}{dt}=r_{N}(t)+s_{P}P(t)$$

$$\frac{dP(t)}{dt}=r_{P}(t)+s_{N}N(t)$$

In this model, the bacterial population consists of two subpopulations, one with a normal phenotype, *N(t)*, and a second with a persister phenotype, *P(t)*. We denote the total bacterial population size by *B(t)*, with *B(t)* = *N(t)*+*P(t)*. The rate of change of the population is determined by the net growth rate of *N* and *P*, *r*_*N*_ and *r*_*P*_, and the switching rate from *N* to *P*, *s*_*N*_, and the switching rate from *P* to *N*, *s*_*P*_. The parameter estimates of the net growth rates are dependent on the AMP concentration A, i.e. *r*_*N*_*(A)* and *r*_*P*_*(A)*. analytical solution of this ODE system [29,77] is

$$B(t)={N(t)+P(t)=c}_{1}u_{1}e^{\lambda_{1}t}+c_{1}u_{2}e^{\lambda_{1}t}+c_{2}v_{1}e^{\lambda_{2}t}+c_{2}v_{2}e^{\lambda_{2}t}$$ (S1)

with

$$\lambda_{1}=\frac{r_{N}+r_{P}-s_{N}-s_{P}-\sqrt{{{(-r}_{N}-r_{P}+s_{N}+s_{P})}^{2}-4(r_{N}r_{P}-r_{P}s_{N}-r_{N}s_{P})}}{2}$$

$$\lambda_{2}=\frac{r_{N}+r_{P}-s_{N}-s_{P}+\sqrt{{{(-r}_{N}-r_{P}+s_{N}+s_{P})}^{2}-4(r_{N}r_{P}-r_{P}s_{N}-r_{N}s_{P})}}{2}$$

$$\overset{´}{u}=\left(\begin{array}{c} \frac{\lambda_{1}-r_{P}+s_{P}}{s_{N}}\\ 1 \end{array}\right)$$

$$\overset{´}{v}=\left(\begin{array}{c} \frac{\lambda_{2}-r_{P}+s_{P}}{s_{N}}\\ 1 \end{array}\right)$$

$$c_{1}=\frac{v_{1}P(t=0)-N(t=0)}{v_{1}{-u}_{1}}$$

$$c_{2}=\frac{{-u}_{1}P(t=0)+N(t=0)}{v_{1}{-u}_{1}}$$

The model was fitted by a least square algorithm, minimizing the residual sum of squares of the data to the prediction. For the starting conditions (*N*(*t* = 0), *P*(*t* = 0)), we assumed that the ratio of *N*/*P* was at the equilibrium predicted by the model without antimicrobials when the exposure to lethal concentrations of AMPs started. *N(t = 0)* and *P(t = 0)* were therefore calculated using the eigenvector $\vec{v}$ that corresponds to the largest eigenvalue of a system without antimicrobials. Here, we assumed that the parameter *r*_*N*_ is equal the net growth rate in absence of antimicrobials, *r*_*N*_ = *ψ*_*max*_. The parameter *ψ*_*max*_ was estimated based on the time-kill curve of bacterial population that grow in absence of antimicrobials (see below and S11 Fig). The eigenvector contains information about the ratio of *N* and *P* for $t\to \infty :\frac{N}{P}=\frac{v_{1}}{v_{2}}$. Resulting, $P(t=0)=\frac{B(t=0)}{1+v_{1}}$ and *N*(*t* = 0) = *B*(*t* = 0) − *P*(*t* = 0). *B*(*t* = 0) was estimated from the data. Confidence intervals were calculated as described above. In a pre-analysis, we used 4 free model parameters that were fitted: *r*_*N*_, *r*_*P*_, *s*_*N*_ and *s*_*P*_. For none of the datasets, the parameter *r*_*P*_ was not significant from 0 (S8 Fig). Therefore, we set the parameter *r*_*P*_ to 0 and fitted the remaining 3 parameters to the data (S9 Fig and S5 Table).

### Tolerance and persistence in terms of model parameters

The measure of tolerance is the slope *m*_*1*_. Komarova and Wodarz [77] showed that the slope can directly be linked to the population model parameters. In our notation,

$$m_{1}={log}_{10}(c_{1}(u_{1}{+v}_{1})e^{\lambda_{1}t})$$ (S2)

Note that the first phase is mainly influenced by *r*_*N*_ (S9 Fig), therefore, *m*_1_ ≈ *r*_*N*_.

Persistent cell numbers at time t were calculated with the analytic solution:

$${P(t)=c}_{1}u_{2}e^{\lambda_{1}t}+c_{2}v_{2}e^{\lambda_{2}t}$$ (S3)

### Pharmacokinetic and pharmacodynamic function

We used the pharmacokinetic function *A*(*t*) = *A*_*max*_*e*^−*k*(*t*−*n*)^, with 8h*n ≤ *t* ≤ 8h*(*n* + 1) and *n* = 0,1,2 … described previously elsewhere [30]. In our simulations, we fixed the decay parameter *k* and varied the drug input *A*_*max*_. To describe the effect of the AMPs on the bacterial population, we used the pharmacodynamic (PD) function *ψ*(*A*) [30,78], with

$$\psi (A)=\psi_{max}-e(A)=\psi_{max}-\frac{(\psi_{max}-\psi_{min}){\left(\frac{A}{MIC}\right)}^{\kappa }}{{\left(\frac{A}{MIC}\right)}^{\kappa }-\frac{\psi_{min}}{\psi_{max}}}$$

The parameter *ψ*_*max*_ describes the net growth rate in absence of antimicrobials (*ψ*_*max*_ = *ψ*(*A* = 0)). The antimicrobial effect *e*(*A*) is dependent on the antimicrobial concentration and is the defined with *ψ*_*max*,_ *ψ*_*min*_, the net growth rate in presence of large amounts of antimicrobials)), with the MIC, the antimicrobial concentration that results in no growth *ψ*(*A* = *MIC*) = 0) and with *κ*, which determines the steepness of the PD curve.

The PD function was fitted to the time-kill curves (S6 Fig), as described by Regoes *et al* [30]. In short, we used log-linear regressions of the time kill curves within the time-points 0h and 1h to estimate the change of the bacterial population over time, i.e. the slopes of the log-linear regression. We fixed the parameter *ψ*_*max*_ and fitted the 3 remaining parameters of the PD function with the *Markov-Chain-Monte-Carlo method*.

### Stochastic simulations

To simulate resistance evolution with stochastic simulations, we expanded a previously developed framework for bacterial population dynamics [9]. The framework models bacterial population dynamics exposed to changing levels of antimicrobials and allows for resistance evolution. In the simulations, the change in population size of a sensitive strain *S*, with *S* = *N*+*P*, and of a resistant strain *R* were described with the following ODE system:

$$\frac{dN(t)}{dt}=\psi_{max}(1-\mu )N(t)(1-\frac{N(t)+P(t)+R(t)}{K}){-s}_{N}N(t)+s_{P}P(t)-e_{N}(A(t))N(t)$$

$$\frac{dP(t)}{dt}=s_{N}N(t)-s_{P}P(t)$$

$$\frac{dR(t)}{dt}=\psi_{max}\mu N(t)(1-\frac{N(t)+P(t)+R(t)}{K}){+\psi }_{max}(1-c)R(1-\frac{N(t)+P(t)+R(t)}{K})-e_{R}(A(t))R(t)$$

Here, the replication rate is assumed to be equal to the maximum net growth rate *ψ*_*max*_. The effect of the antimicrobial *e*_*N*_(*A*) is explained above. Note that we assume that bacteria in class P do not grow and are not affected by antimicrobials. We also assumed that the switching rates are constant. To describe the effect of an antimicrobial on the strain *R*, *e*_*R*_(*A*), we use the same parameter set than with *e*_*N*_(*A*), except for the *MIC*: $\frac{{MIC}_{R}}{{MIC}_{N}}=10$. In the simulations, we differentiated between the following cases: (i) naive bacteria, no persistence (primed -, persisters -), (ii) naive bacteria, persistent subpopulation (primed -, persisters +), (iii) primed bacteria, no persistence (primed +, persisters -), and (iv) primed bacteria, persistent subpopulation (primed +, persisers +). The stochastic simulations were run 1000 times for each antimicrobial, for each case, and for a variety of input antimicrobial concentrations *A*_*max*_. All parameter values are listed in S5 Table. The treatment intensity is described with the input antimicrobial concentrations *A*_*max*_. The simulations were run for t = 7d. Time until clearance and probability of resistance evolution were calculated as the mean of the value over the 1000 simulations.

### Implementation

Statistical testing, simulations and plots were done in R version 3.3.2 [79], using Rstudio version 1.0.143 [80]. We used the following R-packages: (i) for plotting: sfsmisc [81] and plotrix, (ii) for statistical analysis: multcomp, (iii) for fitting the PD function: rjags and (iv) for stochastic simulations: adaptivetau [82]. We used Mathematica version 11.0 [83] to determine the analytical solutions of the population models.

## Supporting information

S1 Fig Bacterial tolerance and persistence change the shape of time-kill curves (Figure A and B adapted from Brauner *et al* [18]. (A) Tolerance is the ability of bacteria to longer survive exposure to antimicrobials due to decrease in susceptibility. Tolerance is quantified as increase in the slope of the time kill curve. (B) Persistence is the phenomenon of a subpopulation being less susceptible to the antimicrobial than the rest of the population. A persistent subpopulation manifests as a biphasic decline of bacterial population when exposed to lethal concentrations of antimicrobials. Here, the population consists predominantly of the less susceptible persistent subpopulation. (C) Together, tolerance and persistence result in biphasic time-kill curves with decreased susceptibility in the first phase.
(TIFF)

S2 Fig A possible explanation for a nonlinear decline in populations could be a decrease in active AMP concentration over time due to degradation. We tested this alternative explanation. As in Fig 1 (main text), we measured the population dynamics of bacteria exposed to 10 x MIC (10 and 20 μg/ml of pexiganan and melittin respectively) (round 1). At the end of the experiment, we sampled the supernatant. In round 2, a fresh bacterial population was exposed to the sampled supernatant. Both panels show survival data of both rounds for melittin and pexiganan, respectively. The trend line depicts the median of the population size at each time-point. We tested differences in population size over time and differences between round one and two for each antimicrobial peptide with an ANOVA and the *p*-values adjusted with the Bonferroni correction for multiple testing. For both, melittin and pexiganan, the population size changed significantly over time, while the differences in population size between round one and two and the interaction between time point and round were not significant (*p* <0.05).
(TIFF)

S3 Fig Diagrammatic representation of (A) the two-state model (see also Fig 5A) and (B) our previously developed framework, which we extended by a persistent class according to Balaban *et al* [29]. Here, bacteria that replicate mutate with a mutation rate *μ* to a more resistant subpopulation *R*.
(TIFF)

S4 Fig Microfluidic device design used for live imaging of priming and killing of *E*. *coli* MG1655 by AMPs. The device consists in two parallel channels with one inlet and one outlet each one and 200 parallel secondary channels that connect with the bacterial confining chambers. The photographs, that were taken with phase contrast at 400X magnification, show close-ups from one of the inlet (A), the main channel with two secondary microchannels (B), and one of the 200 μm confining chamber for bacteria (C). Each chamber square compartment was designed to be similar in size (200 μm) to a microscope field with a magnification of 1000X.
(TIFF)

S5 Fig Quality control of RNA sequencing by evaluating symmetry and distribution of the transcriptome counts, volcano plots showing different degrees of significance (A) and assessing dissimilarities of sample-based Euclidian hierarchical clustering for cells treated with priming concentrations of melittin and pexiganan (B). Datasets are based on RNAseq of three independent biological replicates.
(TIFF)

S6 Fig Parameter values of the two-state model (Fig 2A). (A) The parameters net growth rate of the normal phenotype *rN*, (B) the switching rate from normal to persistent phenotype *sN*, and (C) the switching rate from the persistent phenotype to the normal phenotype *sP* were fitted to the data of primed (orange) and naive (blue) bacteria. The net growth rate of the persistent phenotype *rP* was set to 0 (see also Material and methods). Significant differences between naive (blue) and primed (orange) parameter values are indicated with asterisks. For parameter values, see S5 Table.
(TIFF)

S7 Fig When bacteria are primed with melittin and challenge with pexiganan and the opposite, there are not significant differences between primed and non-primed populations. There is not an effective cross-protection when bacteria were primed with pexiganan and challenged with melittin nor in the opposite direction.
(TIFF)

S8 Fig Net growth rates resulting from the fit of the two-state model with four free parameters. For both melittin and pexiganan, *rP* is not significantly different from 0. Significant differences between naive and primed treatment are indicated with asterisks.
(TIFF)

S9 Fig Pharmacokinetic profile used in our stochastic simulations. The parameter Amax is used to describe treatment intensity.
(TIFF)

S10 Fig Time-kill curves of bacterial populations exposed to AMPs. Time-kill experiments in which naive (blue) and primed (orange) bacteria were exposed to melittin and pexiganan. The AMP doses used in each time-kill experiment are listed in the legend in each plot.
(TIFF)

S11 Fig Pharmacodynamic (PD) curves of *E*. *coli* bacterial population dynamics exposed to AMPs. PD functions of melittin and pexiganan fitted to the data from S10 Fig, respectively. Note that we excluded data points of experiments in which naive bacteria were exposed to melittin (40 μg/ml) from the analysis to ensure the best fit. Parameter values are listed in S5 Table.
(TIFF)

S12 Fig Predictions for different parameter values compared to Fig 10 for the evolution of resistance simulation experiments. (A) Time until clearance and (B) probability of resistance evolution is affected by the AMP decay rate *k* (dashed lines: *k* = 0.1, solid lines: *k* = 0.3). (C) Time 5 until clearance and (D) probability of resistance evolution is also affected by the mutation rate (in the curves with dashed lines: μ = 10^–11^, solid lines: μ = 10^–9^). All simulations are based on melittin data. We simulated primed bacteria with heterogeneous population consisting of *N* and *P* subpopulations (primed +, persisters +), and naive bacteria with heterogeneous subpopulation (primed -, persisters +). In addition, we simulated dynamics without persistence for both primed 10 and naive bacteria (primed +, persisters - and primed -, persisters–). In (A) and (C), no clearance (grey area) means that simulated treatment could not reduce bacteria population < 1 cell within 7 days of treatment. All parameter values used in the mathematical model are listed in S5 Table.
(TIFF)

S13 Fig Two-state model predicts biphasic decline depending on the model parameter values. If not varied, *rN* = -10, *rP* = 0, *sN* = 0.001, and *sP* = 1. Tolerance, i.e. the slope *m1* is mainly influenced by *rN*, while the levels of persistence is influenced by all four parameters.
(TIFF)

S1 Table Minimal inhibitory concentration (MIC) of *E*. *coli* MG1655 for the two antimicrobial peptides melittin and pexiganan and the antibiotic ciprofloxacin used in this work. The MIC values were determined as reference for the experiments. In addition, the MIC was also determined after priming (0.1xMIC), and for the surviving fraction after exposing bacteria to the final challenge or triggering of the priming response (10xMIC). The unchanged MIC values indicate that the enhanced survival due to the peptides treatments is consequence of phenotypic changes but not selection of mutants in the treated populations.
(TIFF)

S2 Table Slopes of fitting the biphasic function with the slopes *m1* and *m2* to the data on a log10 scale. Asterisks indicate statistically significant differences between the slopes of primed and naive population counts for each antimicrobial.
(TIFF)

S3 Table Results of fitting the two-state model to bacterial population dynamics in the presence of melittin and pexiganan and the classic exponential population growth model. (TIFF)

S4 Table Characterization of the priming response to pexiganan and melittin (0.1xMIC, 30-minute treatment) obtained from transcriptional profiling determined by RNAseq (see excel file S4 Table containing up and down regulated transcripts by melittin and pexiganan at priming concentrations during 30 minutes of exposure). (XLSX)

S5 Table Parameter values used as input for the computer stochastic simulations. (TIFF)

S6 Table Strains and plasmids used in this work and their relevant phenotypes. (TIFF)
