# Supplementary figures and images for: Bacteria primed by antimicrobial peptides develop tolerance and persist
Source: PLoS Pathog. 2021 Mar 31;17(3):e1009443. doi: 10.1371/journal.ppat.1009443 (PMC8041211; doi:10.1371/journal.ppat.1009443)

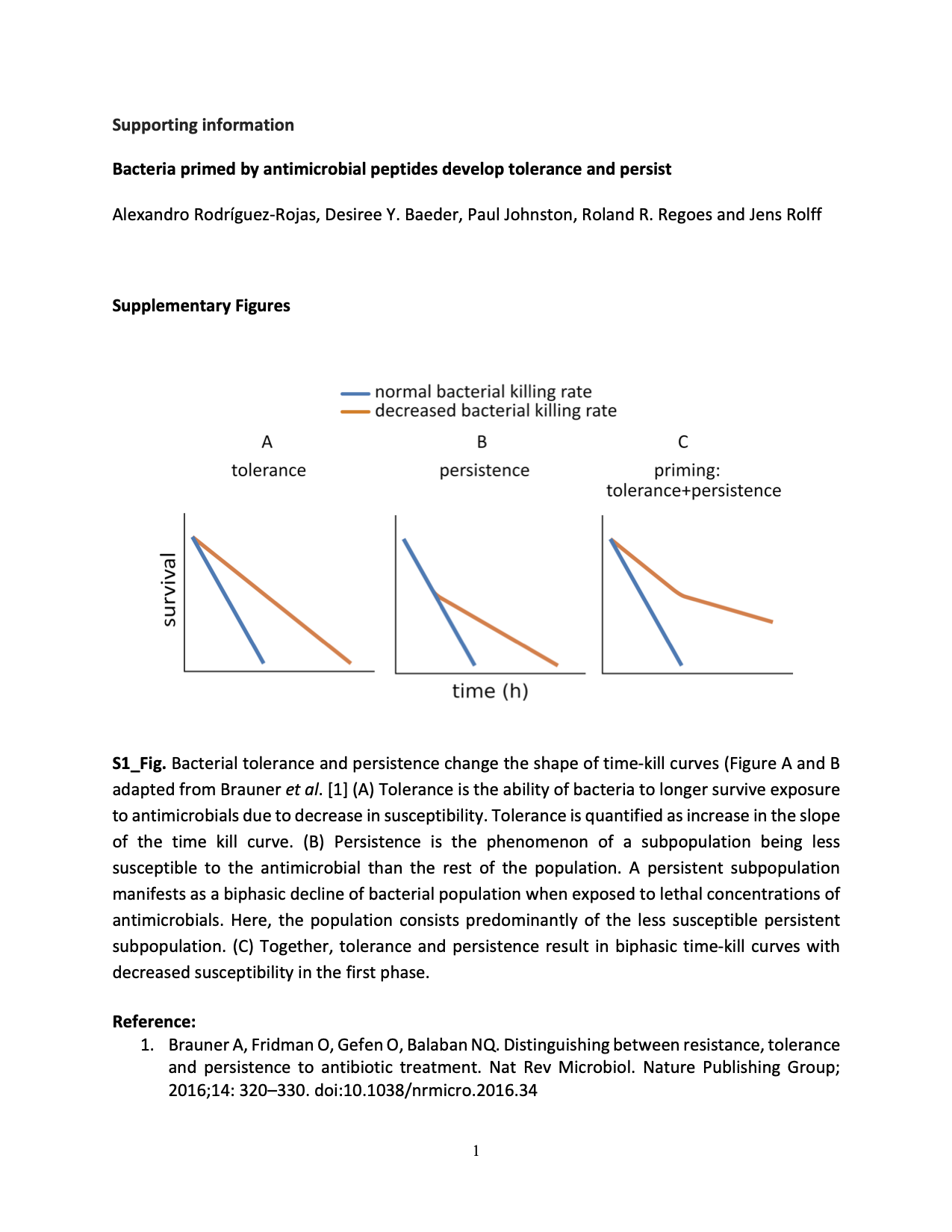

Supplement: S1 Fig — (A) Tolerance is the ability of bacteria to longer survive exposure to antimicrobials due to decrease in susceptibility. Tolerance is quantified as increase in the slope of the time kill curve. (B) Persistence is the phenomenon of a subpopulation being less susceptible to the antimicrobial than the rest of the population. A persistent subpopulation manifests as a biphasic decline of bacterial population when exposed to lethal concentrations of antimicrobials. Here, the population consists predominantly of the less susceptible persistent subpopulation. (C) Together, tolerance and persistence result in biphasic time-kill curves with decreased susceptibility in the first phase. (TIFF) [file ppat.1009443.s001.tiff]

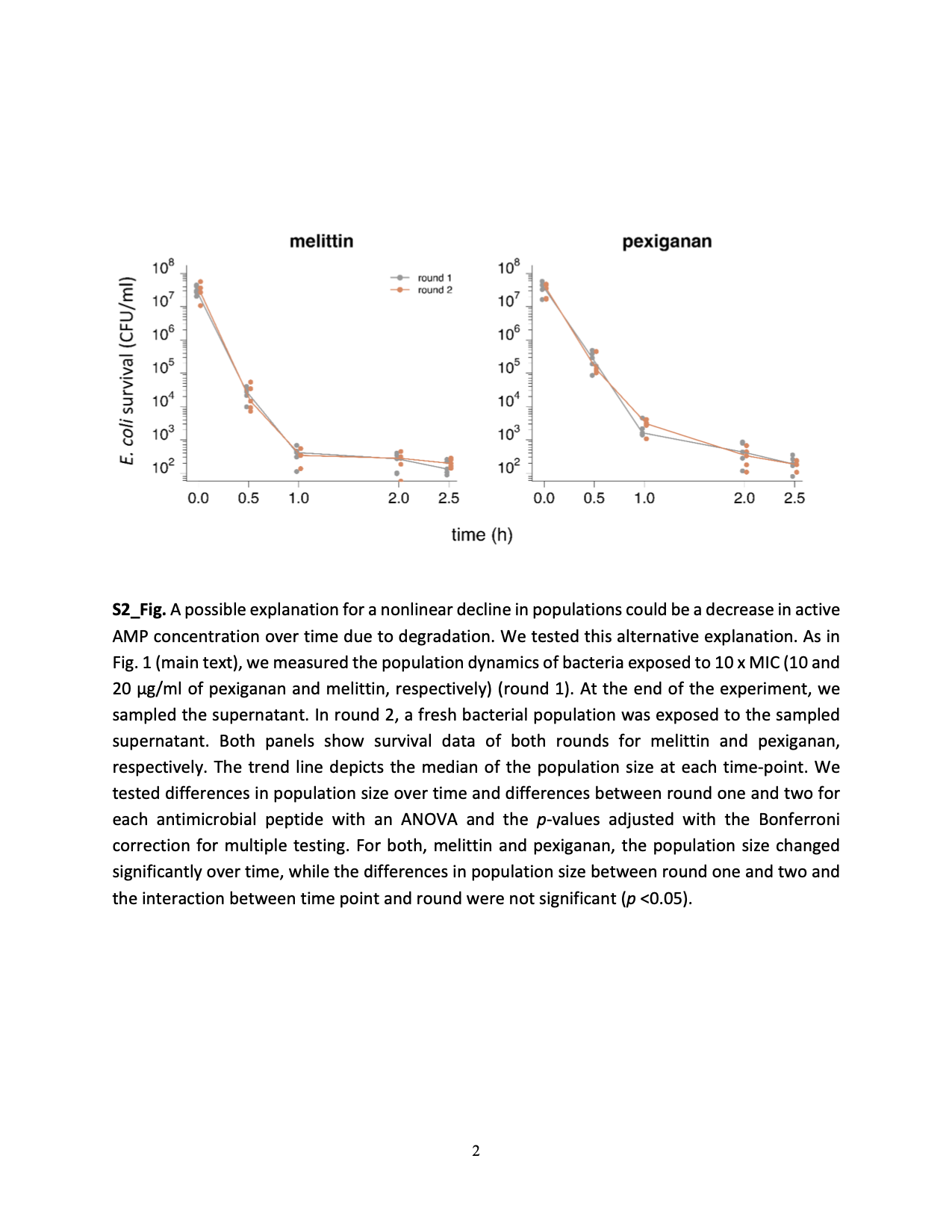

Supplement: S2 Fig — We tested this alternative explanation. As in Fig 1 (main text), we measured the population dynamics of bacteria exposed to 10 x MIC (10 and 20 μg/ml of pexiganan and melittin respectively) (round 1). At the end of the experiment, we sampled the supernatant. In round 2, a fresh bacterial population was exposed to the sampled supernatant. Both panels show survival data of both rounds for melittin and pexiganan, respectively. The trend line depicts the median of the population size at each time-point. We tested differences in population size over time and differences between round one and two for each antimicrobial peptide with an ANOVA and the p-values adjusted with the Bonferroni correction for multiple testing. For both, melittin and pexiganan, the population size changed significantly over time, while the differences in population size between round one and two and the interaction between time point and round were not significant (p <0.05). (TIFF) [file ppat.1009443.s002.tiff]

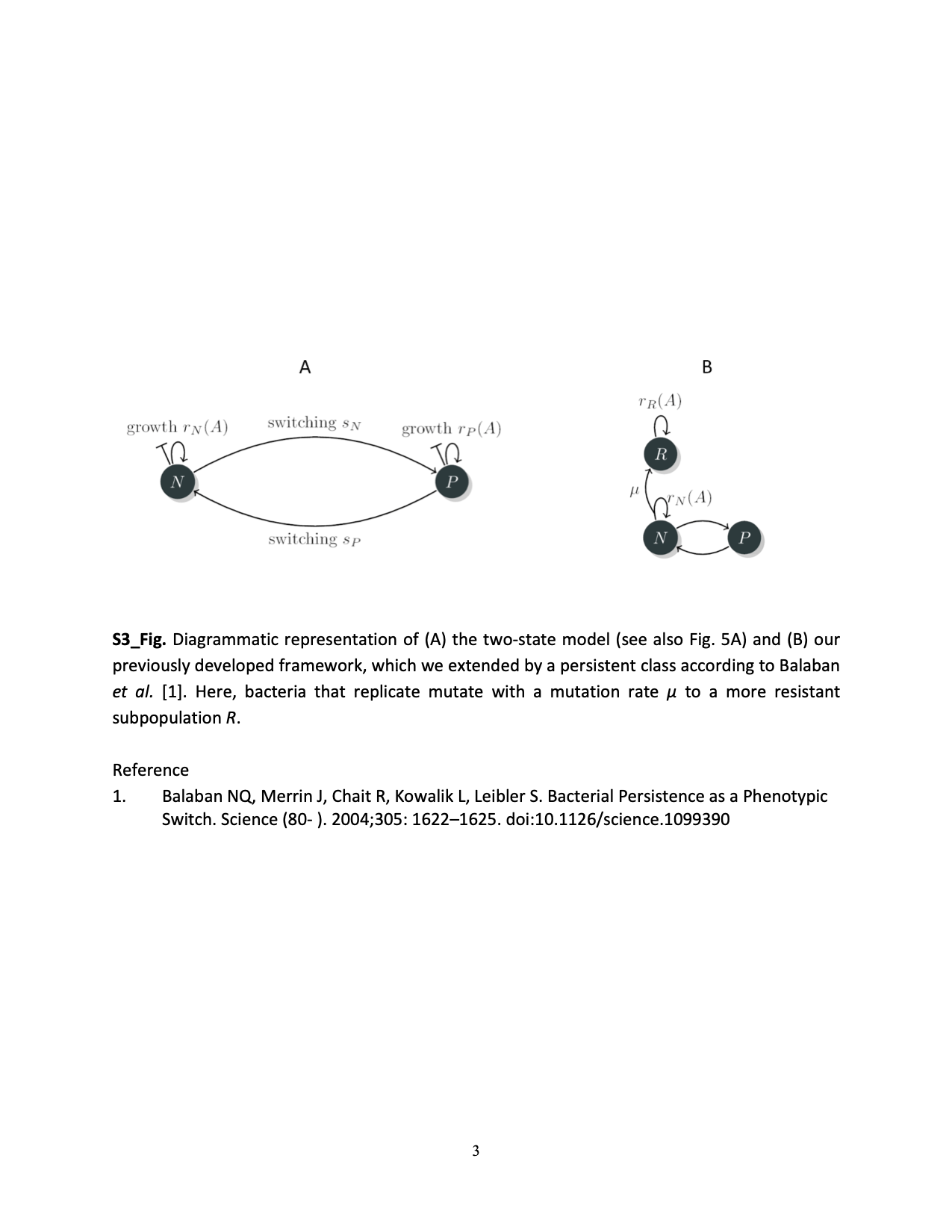

Supplement: S3 Fig — Here, bacteria that replicate mutate with a mutation rate μ to a more resistant subpopulation R. (TIFF) [file ppat.1009443.s003.tiff]

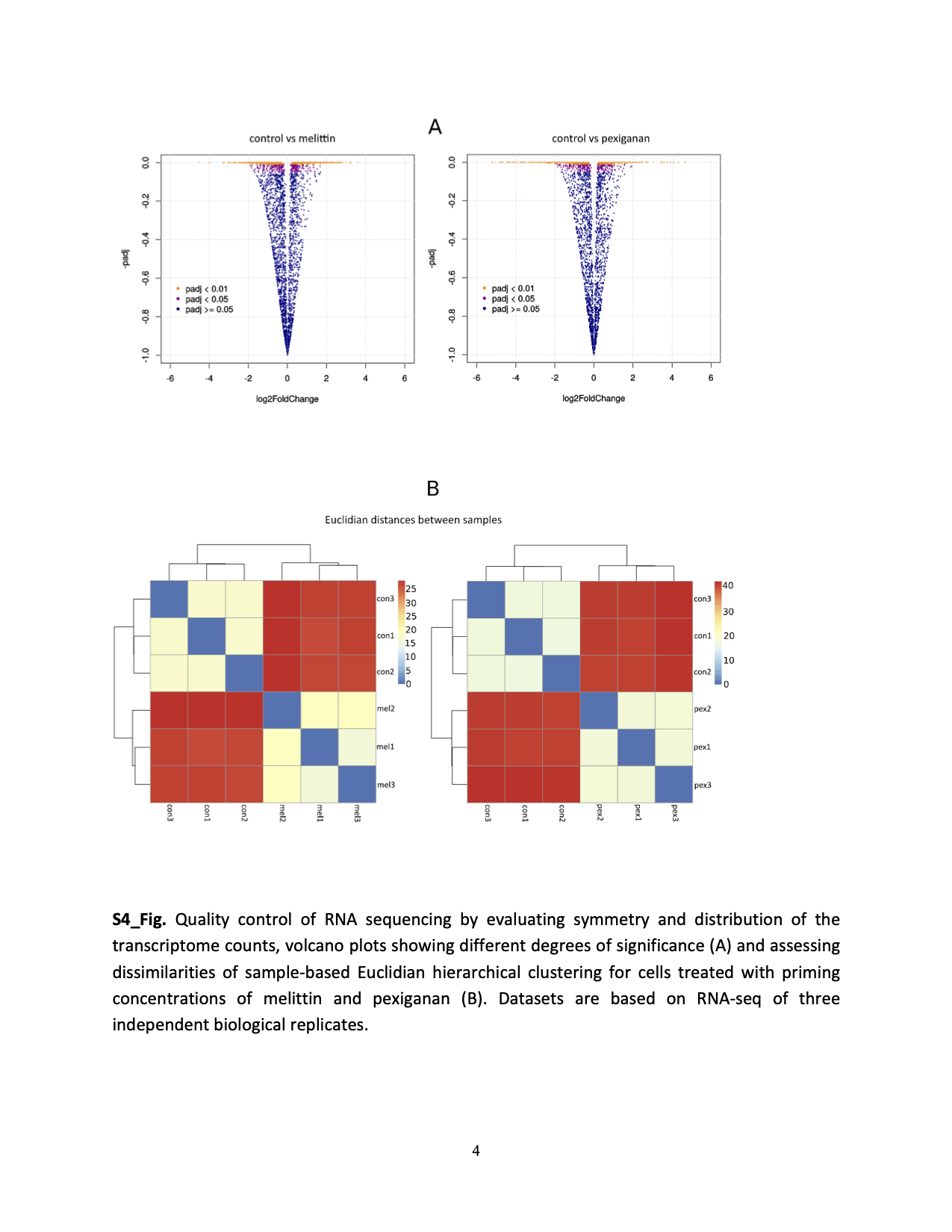

Supplement: S4 Fig — The device consists in two parallel channels with one inlet and one outlet each one and 200 parallel secondary channels that connect with the bacterial confining chambers. The photographs, that were taken with phase contrast at 400X magnification, show close-ups from one of the inlet (A), the main channel with two secondary microchannels (B), and one of the 200 μm confining chamber for bacteria (C). Each chamber square compartment was designed to be similar in size (200 μm) to a microscope field with a magnification of 1000X. (TIFF) [file ppat.1009443.s004.tiff]

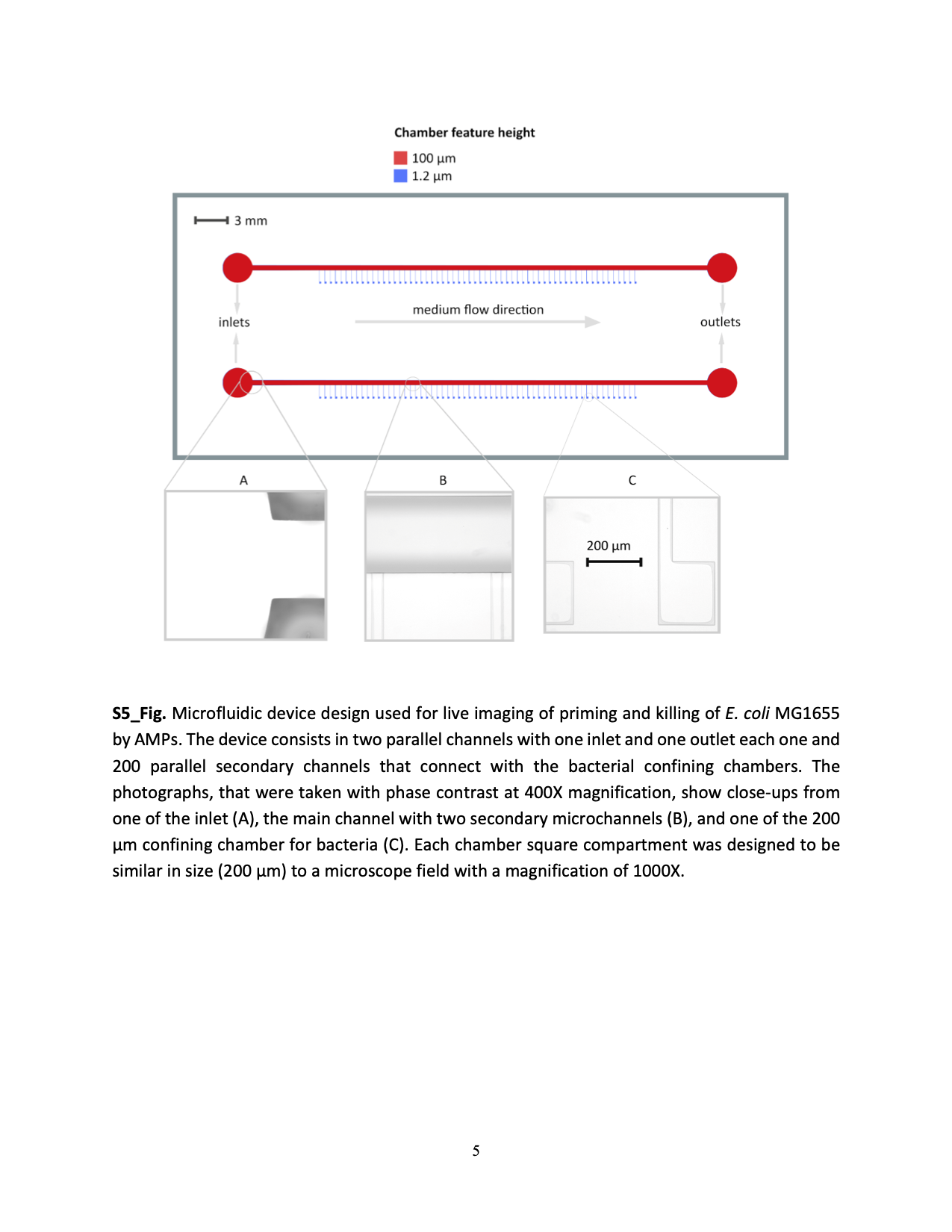

Supplement: S5 Fig — Datasets are based on RNAseq of three independent biological replicates. (TIFF) [file ppat.1009443.s005.tiff]

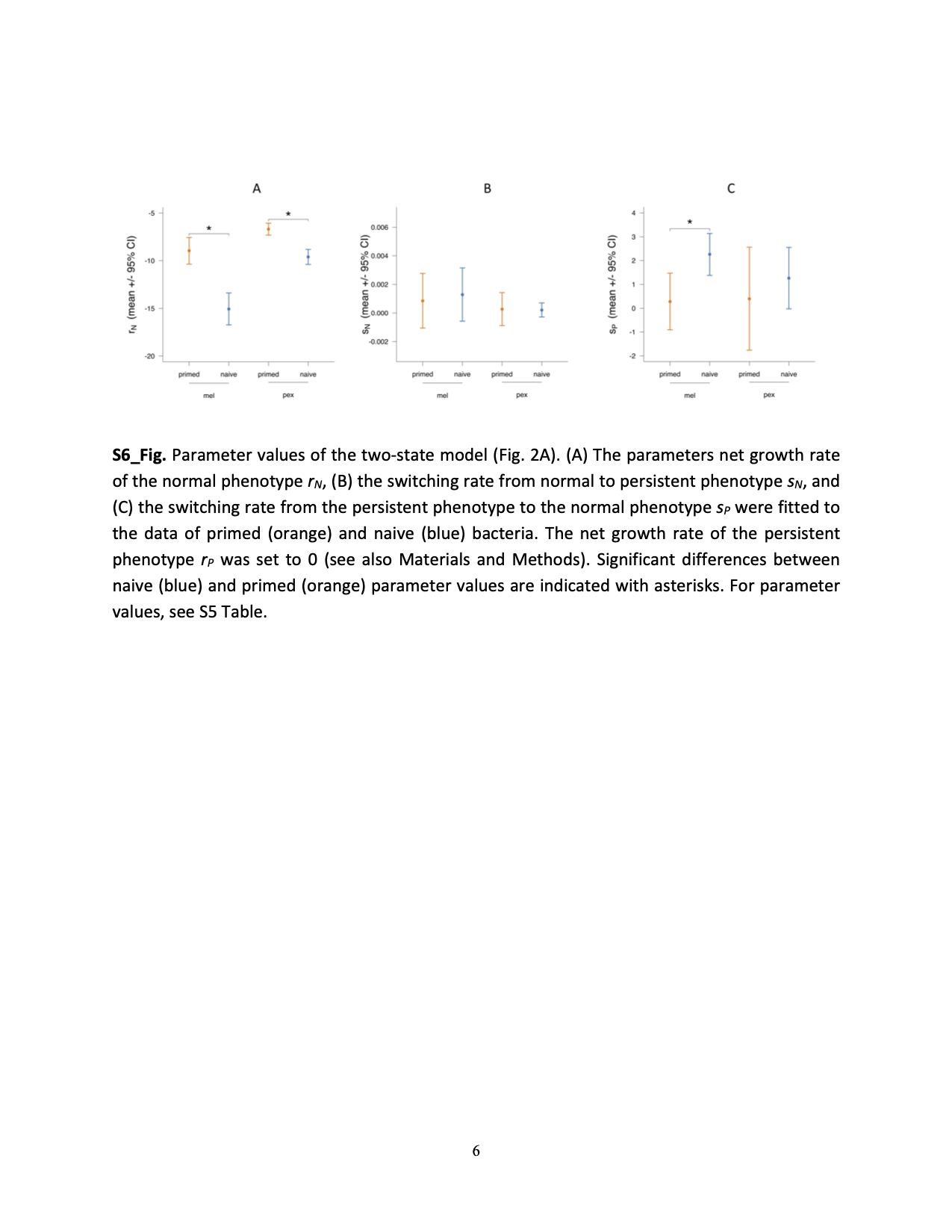

Supplement: S6 Fig — (A) The parameters net growth rate of the normal phenotype rN, (B) the switching rate from normal to persistent phenotype sN, and (C) the switching rate from the persistent phenotype to the normal phenotype sP were fitted to the data of primed (orange) and naive (blue) bacteria. The net growth rate of the persistent phenotype rP was set to 0 (see also Material and methods). Significant differences between naive (blue) and primed (orange) parameter values are indicated with asterisks. For parameter values, see S5 Table. (TIFF) [file ppat.1009443.s006.tiff]

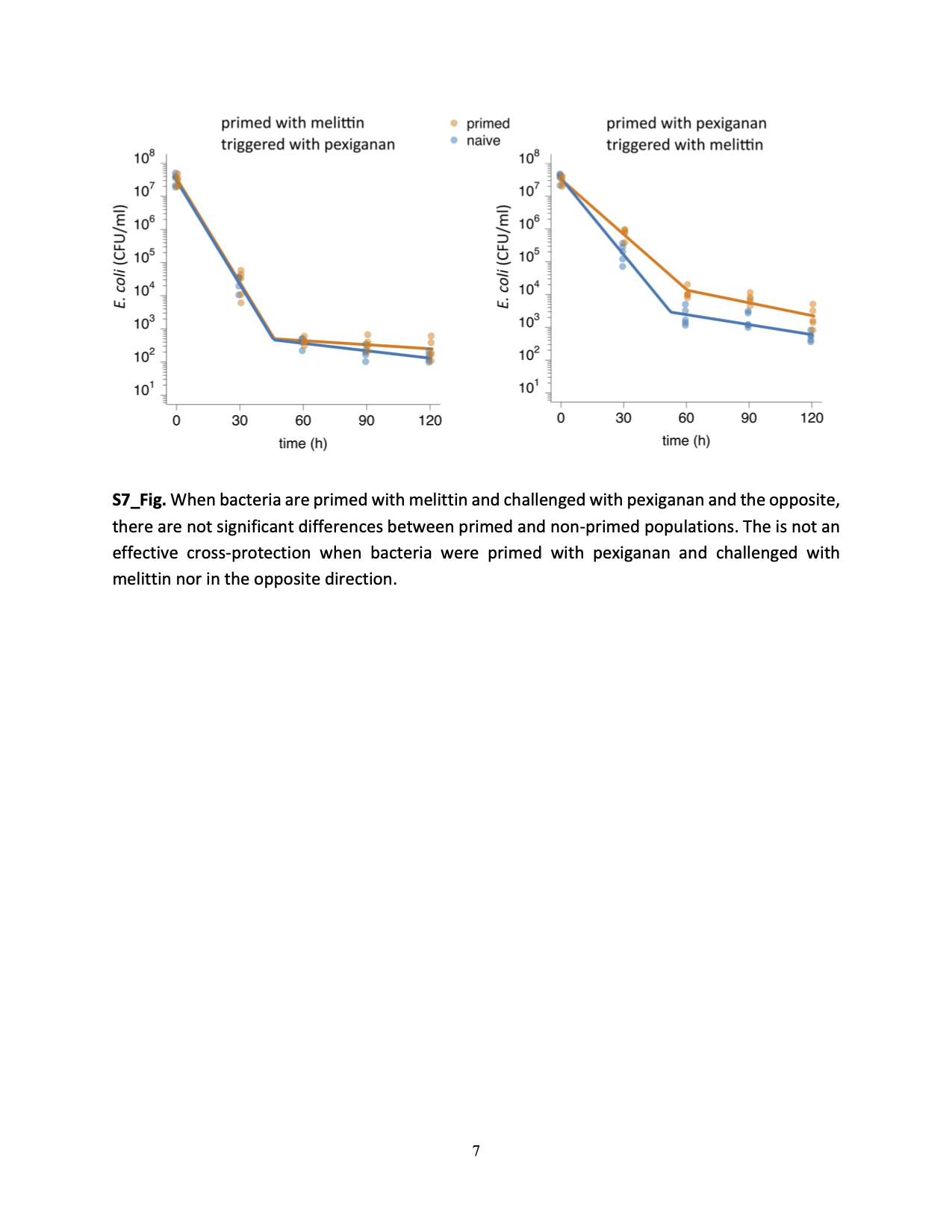

Supplement: S7 Fig — There is not an effective cross-protection when bacteria were primed with pexiganan and challenged with melittin nor in the opposite direction. (TIFF) [file ppat.1009443.s007.tiff]

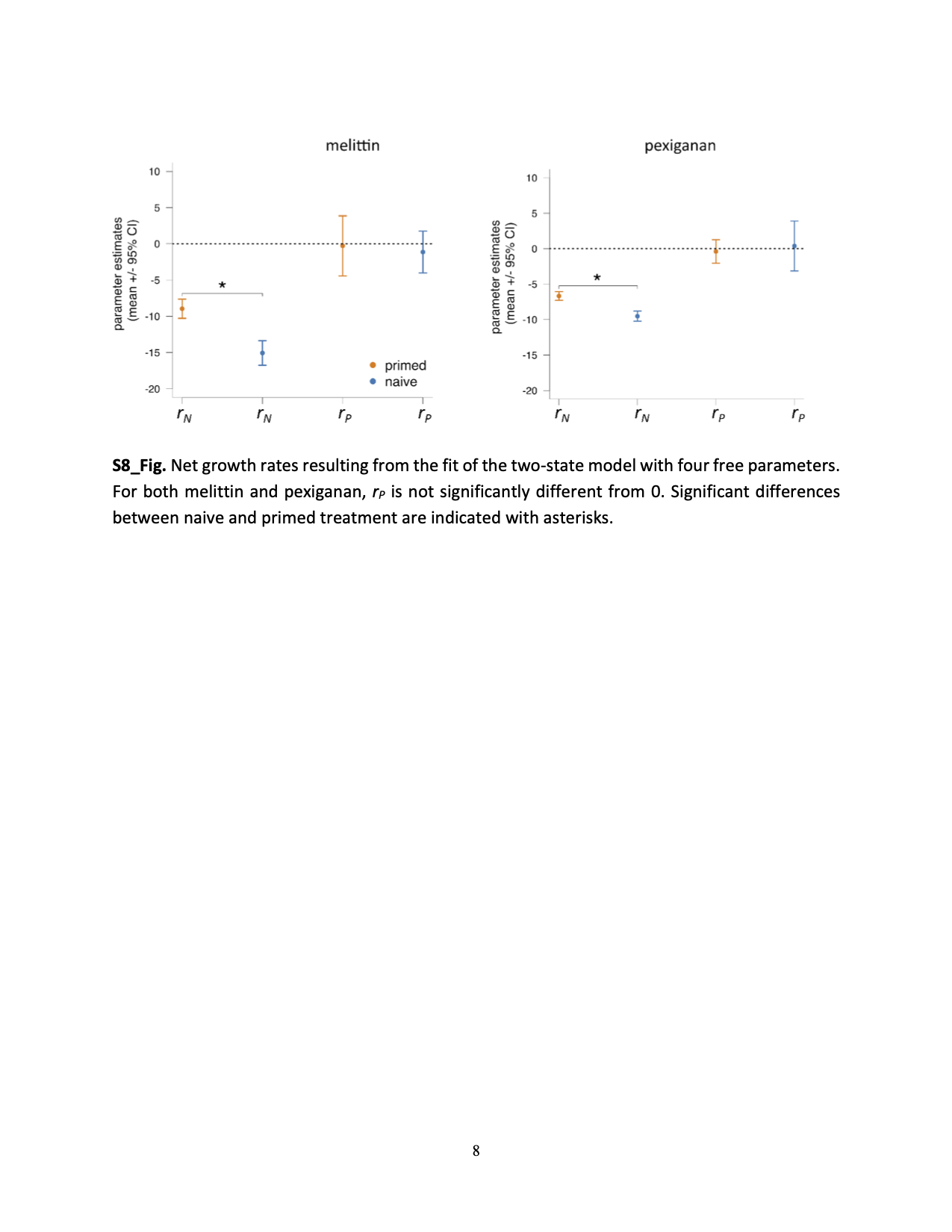

Supplement: S8 Fig — For both melittin and pexiganan, rP is not significantly different from 0. Significant differences between naive and primed treatment are indicated with asterisks. (TIFF) [file ppat.1009443.s008.tiff]

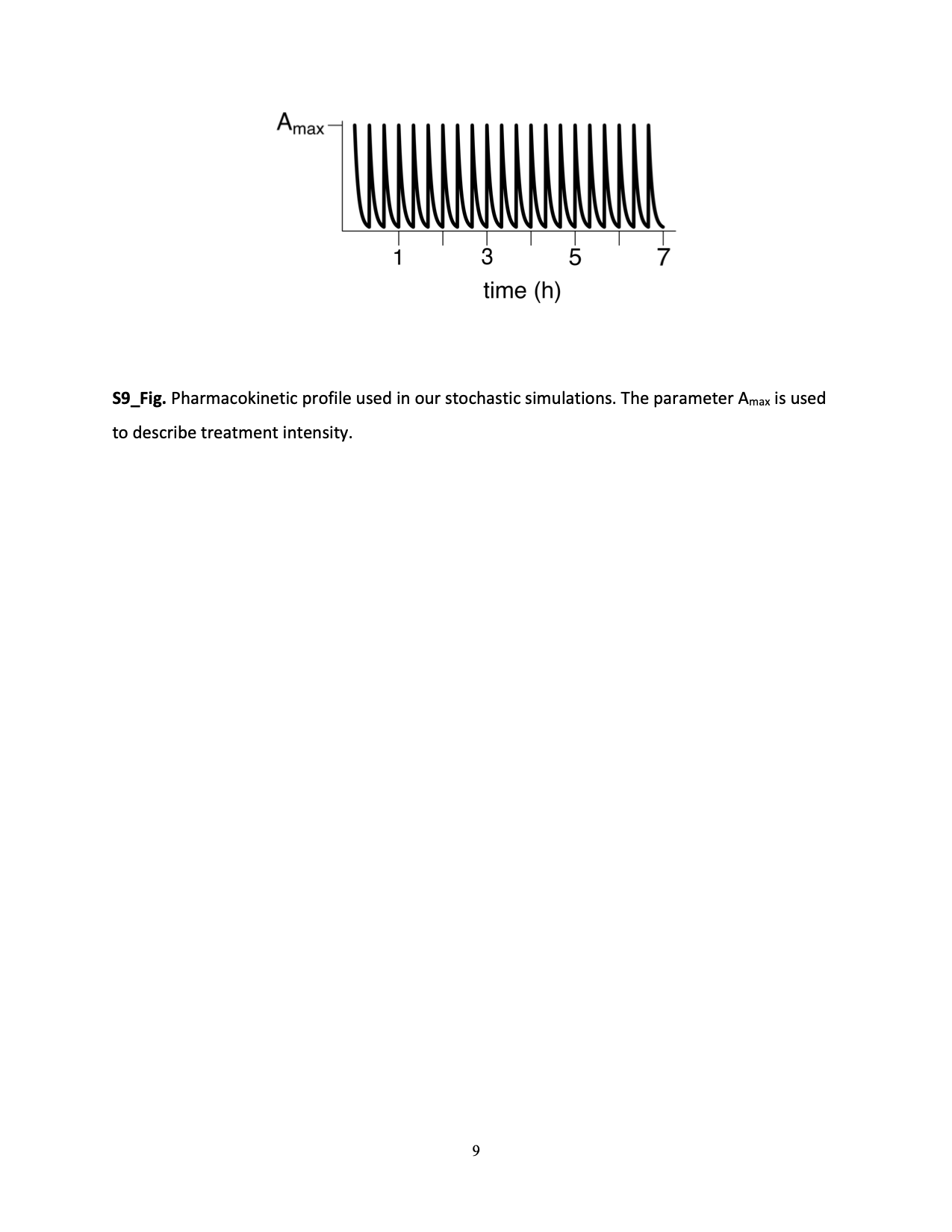

Supplement: S9 Fig — The parameter Amax is used to describe treatment intensity. (TIFF) [file ppat.1009443.s009.tiff]

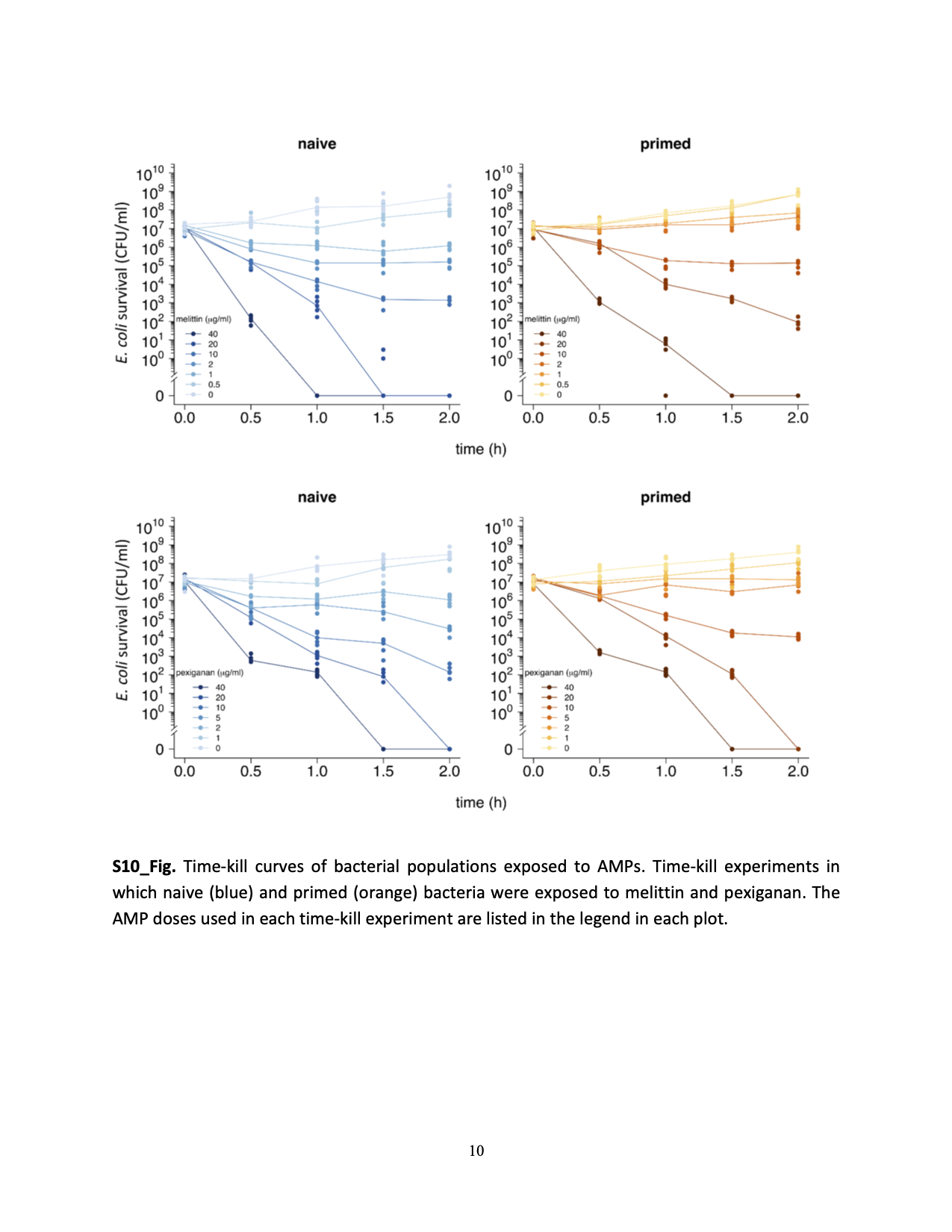

Supplement: S10 Fig — Time-kill experiments in which naive (blue) and primed (orange) bacteria were exposed to melittin and pexiganan. The AMP doses used in each time-kill experiment are listed in the legend in each plot. (TIFF) [file ppat.1009443.s010.tiff]

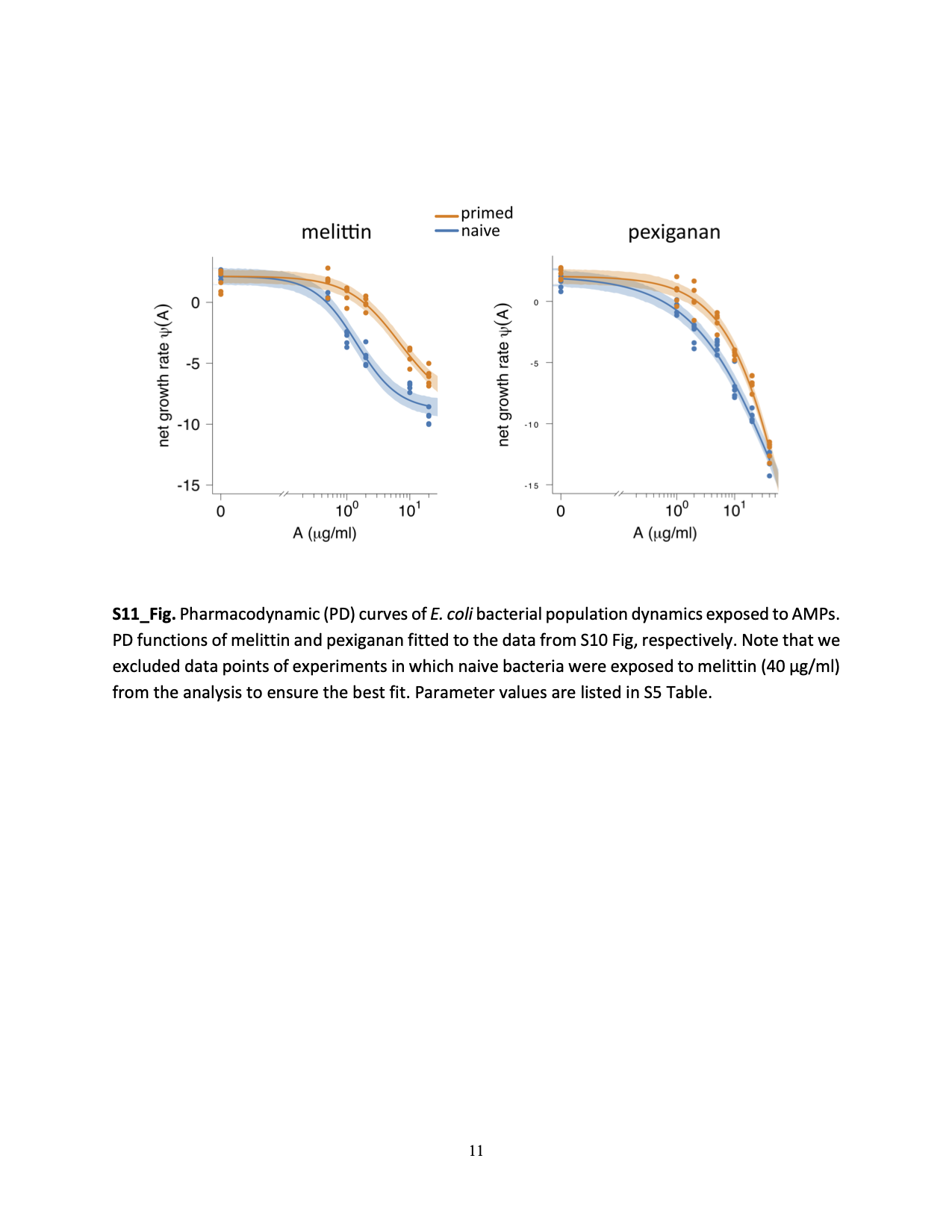

Supplement: S11 Fig — PD functions of melittin and pexiganan fitted to the data from S10 Fig, respectively. Note that we excluded data points of experiments in which naive bacteria were exposed to melittin (40 μg/ml) from the analysis to ensure the best fit. Parameter values are listed in S5 Table. (TIFF) [file ppat.1009443.s011.tiff]

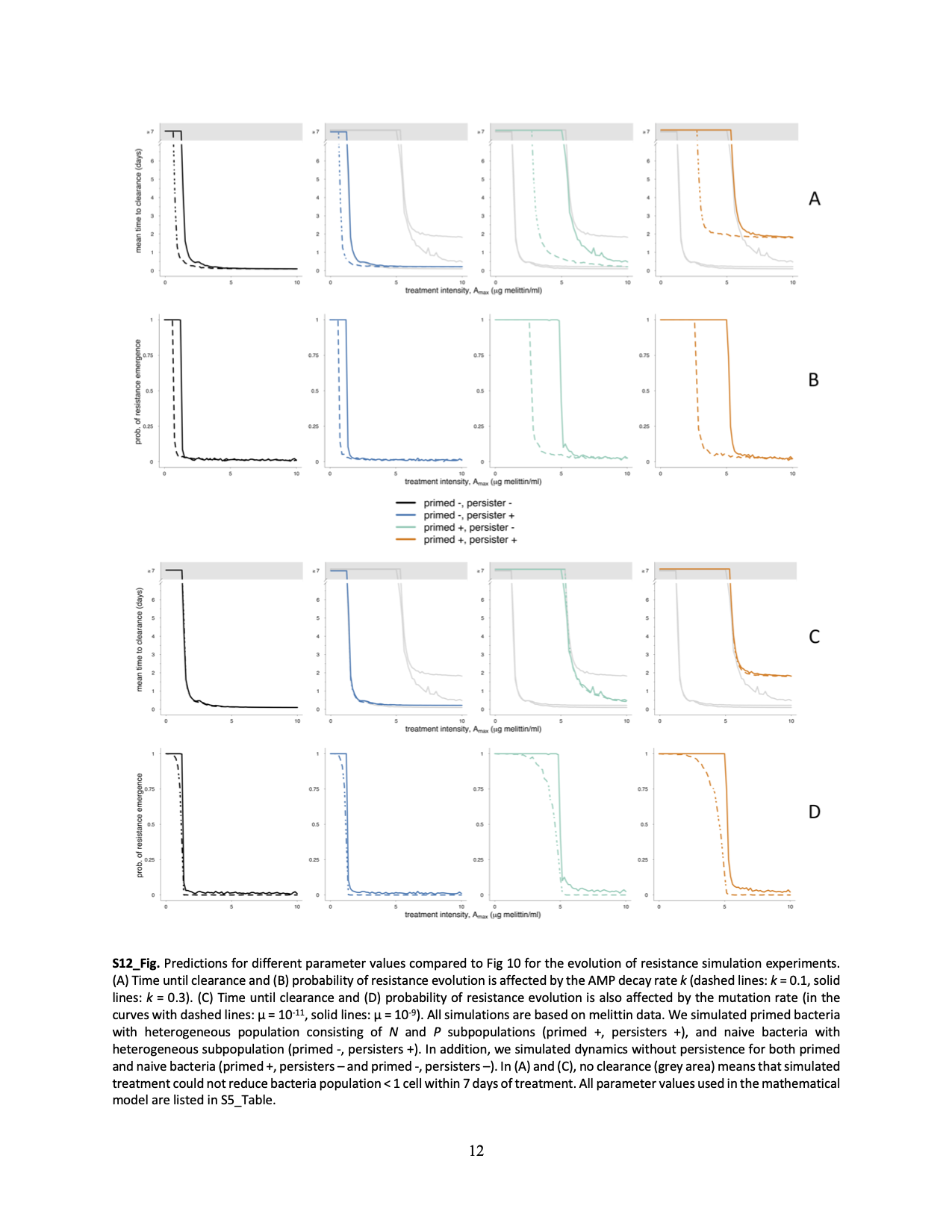

Supplement: S12 Fig — (A) Time until clearance and (B) probability of resistance evolution is affected by the AMP decay rate k (dashed lines: k = 0.1, solid lines: k = 0.3). (C) Time 5 until clearance and (D) probability of resistance evolution is also affected by the mutation rate (in the curves with dashed lines: μ = 10–11, solid lines: μ = 10–9). All simulations are based on melittin data. We simulated primed bacteria with heterogeneous population consisting of N and P subpopulations (primed +, persisters +), and naive bacteria with heterogeneous subpopulation (primed -, persisters +). In addition, we simulated dynamics without persistence for both primed 10 and naive bacteria (primed +, persisters - and primed -, persisters–). In (A) and (C), no clearance (grey area) means that simulated treatment could not reduce bacteria population < 1 cell within 7 days of treatment. All parameter values used in the mathematical model are listed in S5 Table. (TIFF) [file ppat.1009443.s012.tiff]

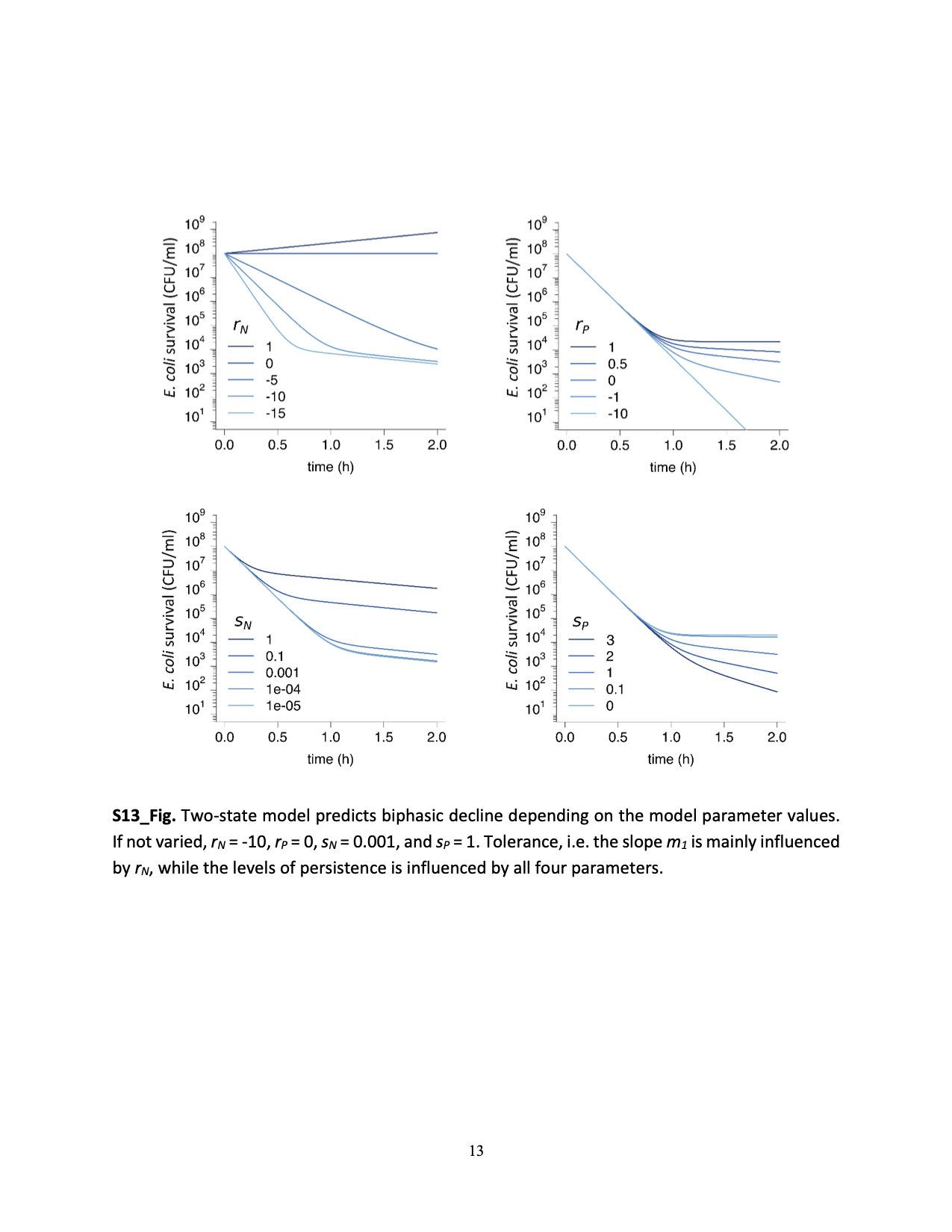

Supplement: S13 Fig — If not varied, rN = -10, rP = 0, sN = 0.001, and sP = 1. Tolerance, i.e. the slope m1 is mainly influenced by rN, while the levels of persistence is influenced by all four parameters. (TIFF) [file ppat.1009443.s013.tiff]

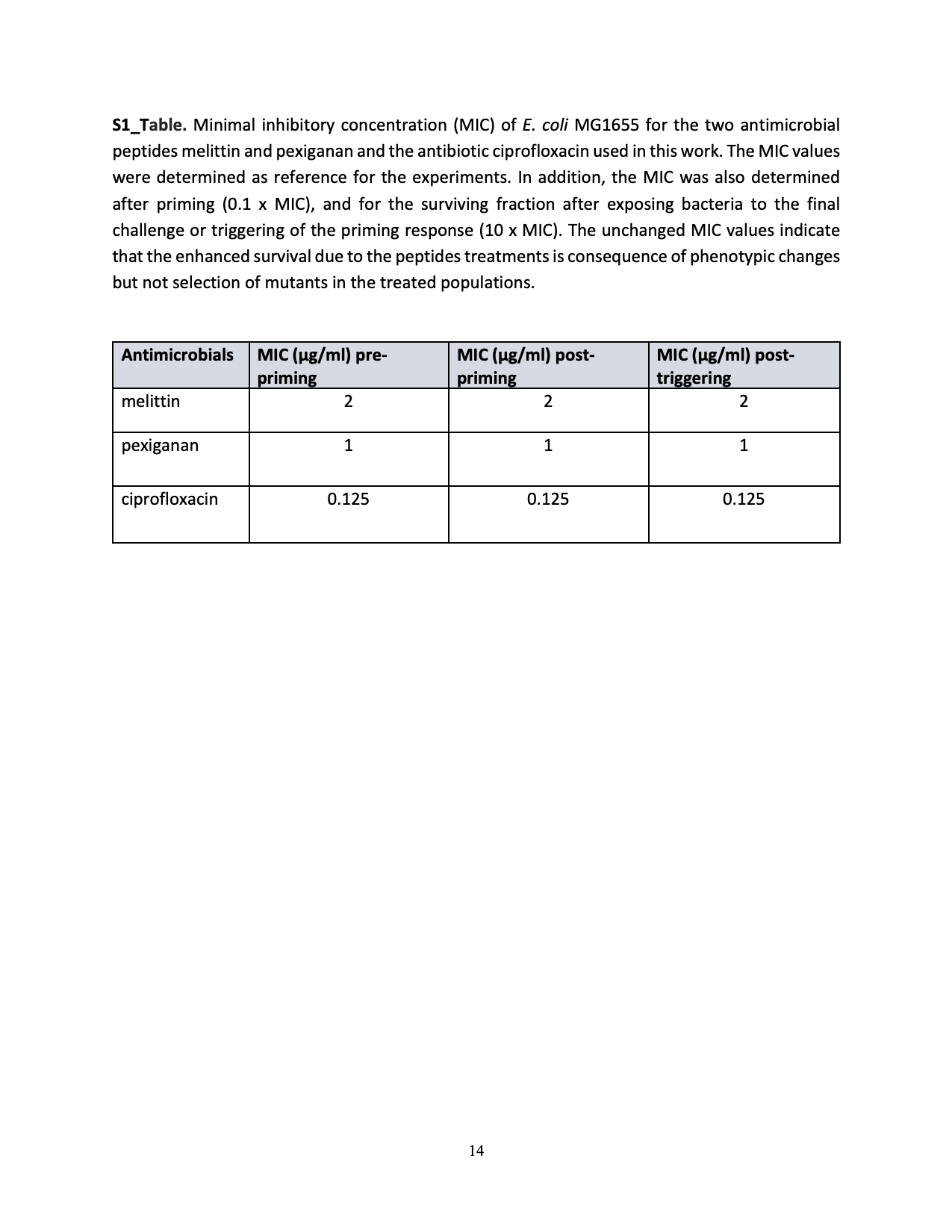

Supplement: S1 Table — The MIC values were determined as reference for the experiments. In addition, the MIC was also determined after priming (0.1xMIC), and for the surviving fraction after exposing bacteria to the final challenge or triggering of the priming response (10xMIC). The unchanged MIC values indicate that the enhanced survival due to the peptides treatments is consequence of phenotypic changes but not selection of mutants in the treated populations. (TIFF) [file ppat.1009443.s014.tiff]

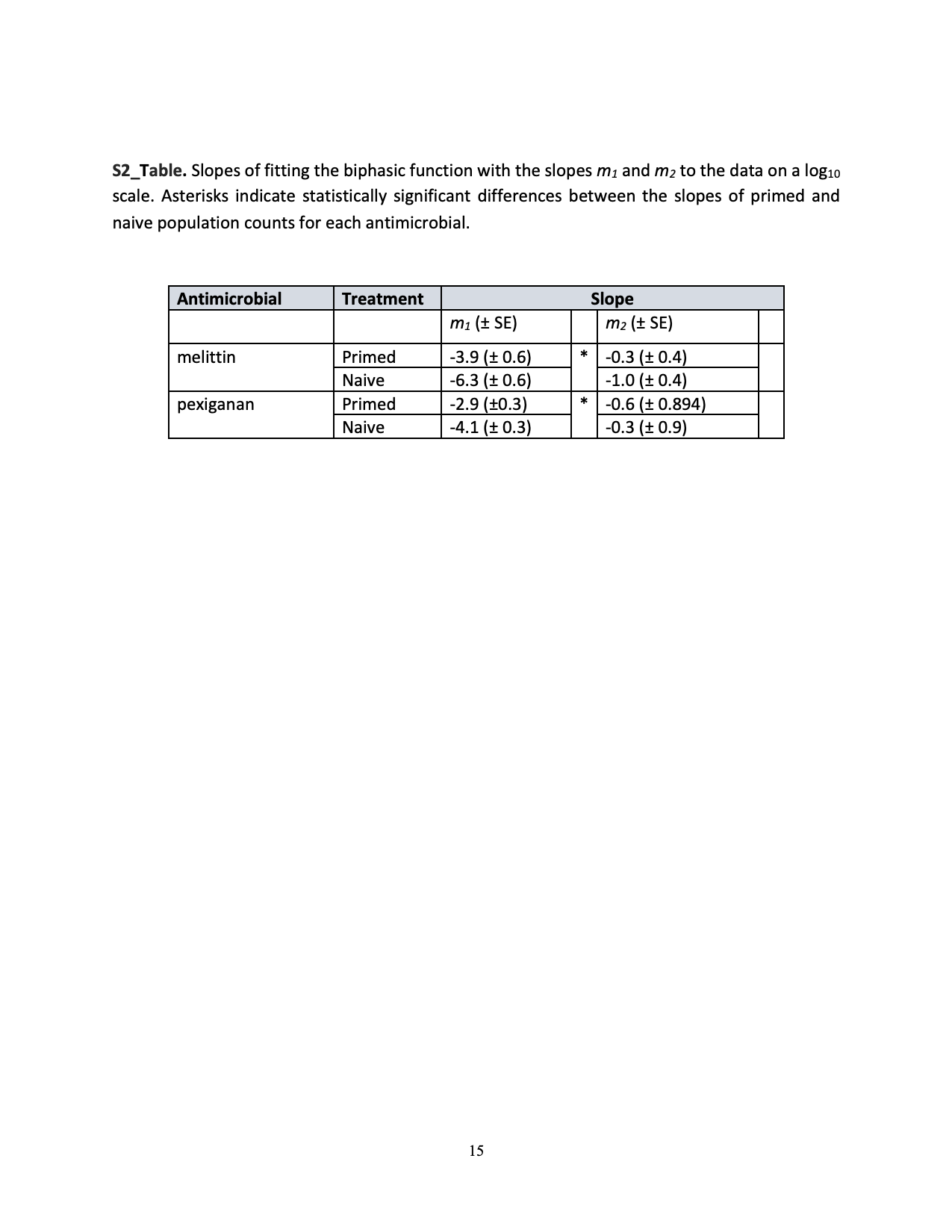

Supplement: S2 Table — Asterisks indicate statistically significant differences between the slopes of primed and naive population counts for each antimicrobial. (TIFF) [file ppat.1009443.s015.tiff]

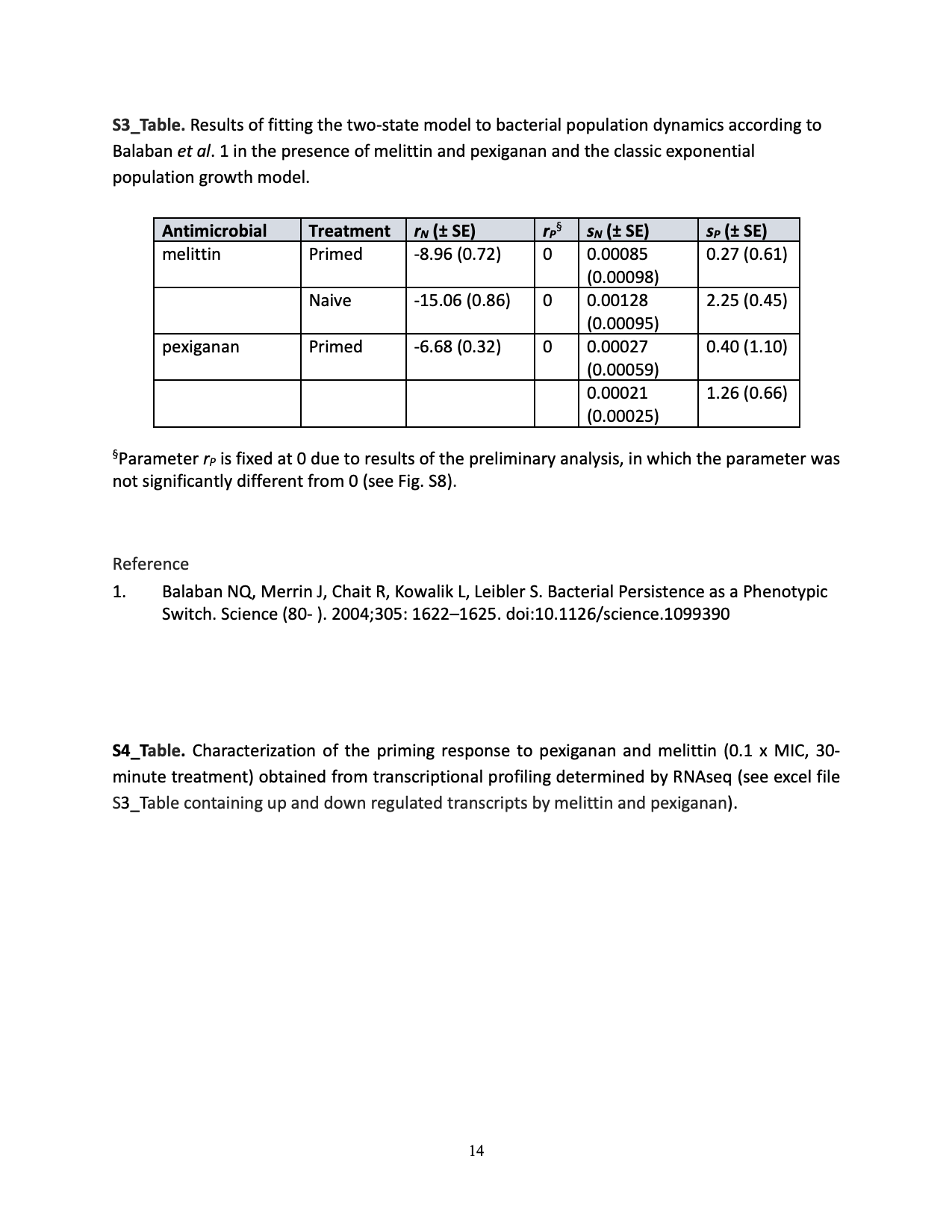

Supplement: S3 Table — (TIFF) [file ppat.1009443.s016.tiff]

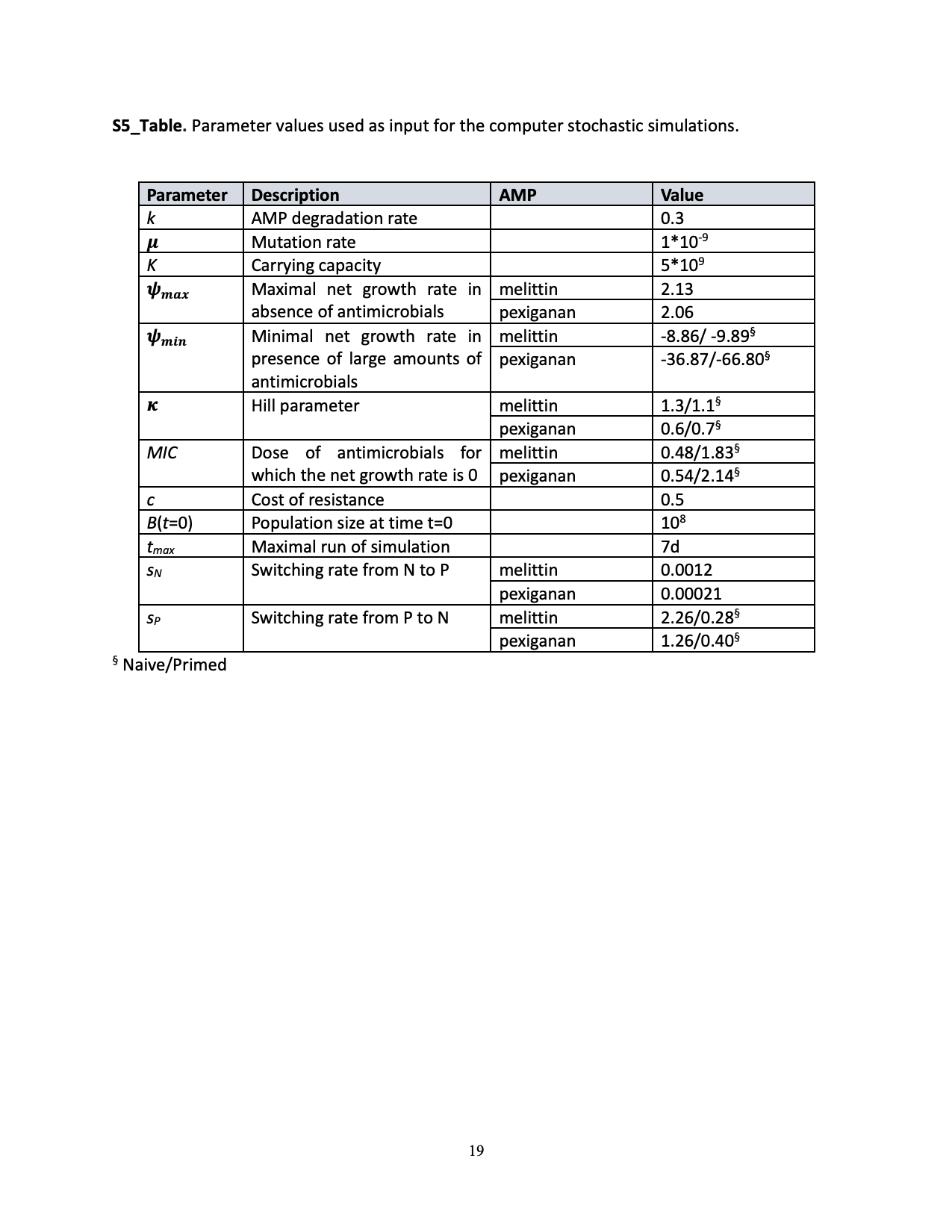

Supplement: S5 Table — (TIFF) [file ppat.1009443.s018.tiff]

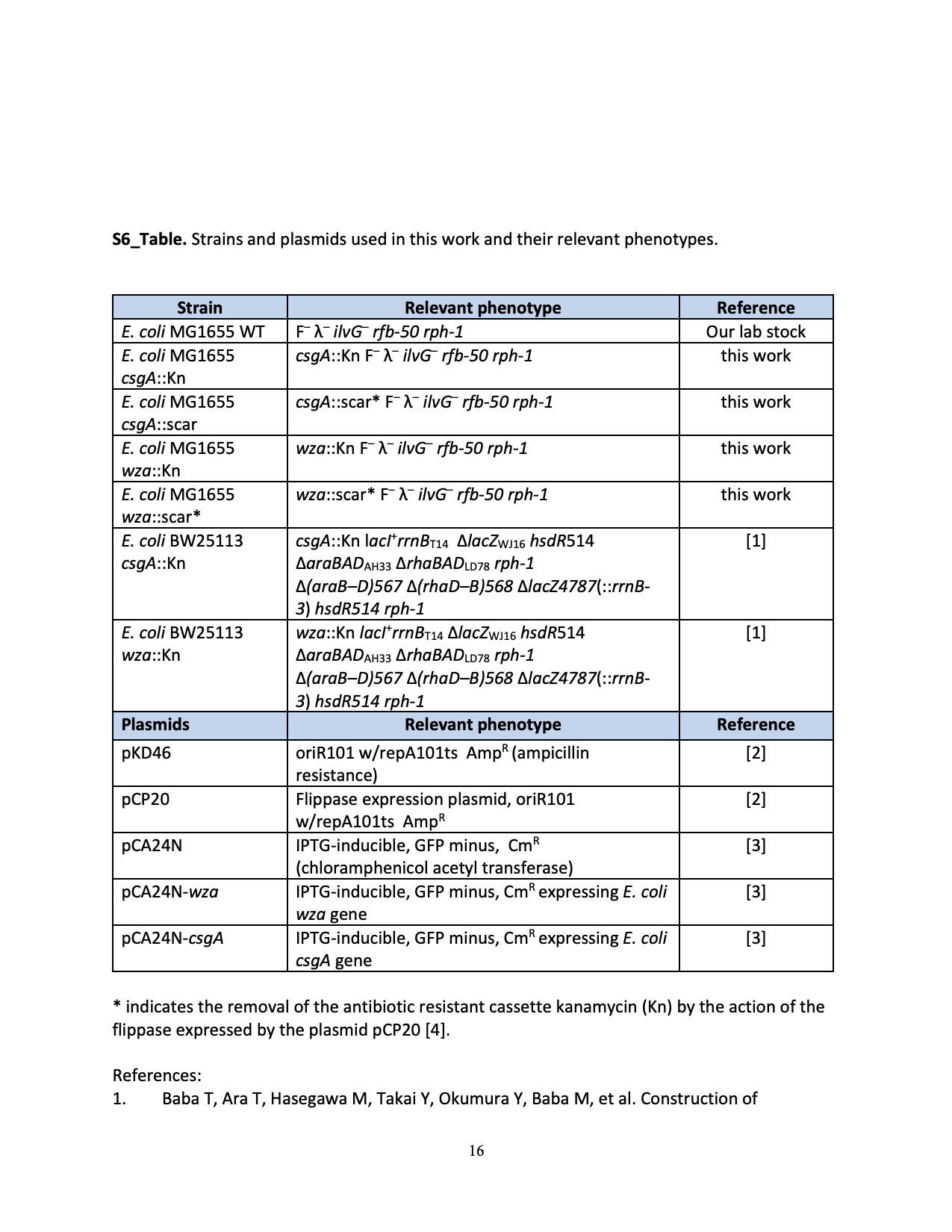

Supplement: S6 Table — (TIFF) [file ppat.1009443.s019.tiff]
